# Combination of Sample Preservation Approaches and DNA Extraction Methods for Long‐Read Sequencing of Nudibranchs' Genomes

**DOI:** 10.1002/ece3.71262

**Published:** 2025-04-15

**Authors:** Inés Alberola‐Mora, Oleanna Guerra‐Font, Omar Daniel Espinoza‐Calderón, Carles Galià‐Camps, Mária Džunková

**Affiliations:** ^1^ Institute for Integrative Systems Biology (I2SysBio) University of Valencia and Spanish National Research Council (CSIC) Valencia Spain; ^2^ Department of Genetics, Microbiology and Statistics, Faculty of Biology University of Barcelona Barcelona Spain; ^3^ Institut de Recerca de la Biodiversitat (IRBio), faculty of Biology University of Barcelona Barcelona Spain; ^4^ Blanes Centre for Advanced Studies Spanish National Research Council (CSIC) Blanes Spain

**Keywords:** DNA extraction, high molecular weight DNA, long‐read sequencing, Nudibranchia, sample preservation

## Abstract

With the increasing interest in whole genome sequencing of eukaryotes, it is becoming evident that selecting the most suitable high molecular weight DNA extraction method is crucial for maximizing the benefits of long‐read technologies. However, the DNA of many species cannot be processed immediately at the sampling site due to the remoteness of the location, necessitating tissue preservation that may affect DNA fragment size. This study aimed to identify the most suitable combination of four tissue preservation approaches and six DNA extraction methods to ensure high molecular weight DNA. A single *Peltodoris atromaculata* (Nudibranchia) specimen was sliced into ∼30 mg sub‐samples, ensuring consistency across 24 preservation‐extraction combinations processed in triplicates. Samples were either stored at 4°C, dried at room temperature, flash‐frozen in liquid nitrogen, or preserved in ethanol and stored at −20°C. Afterward, they were processed using five commercially available kits specific for high molecular weight DNA extraction, as well as a custom DNA extraction protocol. Three aspects of DNA quality were evaluated: total yield, fragment size distribution, and availability of DNA for amplification. Most preservation‐extraction combinations yielded optimal results in only some of the three DNA quality aspects. We identified six combinations suitable for long‐read sequencing: a custom CTAB‐based extraction protocol applied to frozen samples, Wizard (Promega) and Nanobind (PacBio) kits for both frozen and ethanol‐preserved samples, and the ethanol preservation paired with Monarch (NEB) kits. The suitability of the six selected combinations was confirmed by PacBio sequencing, producing a total yield of 3.6 Gbp (3.2x estimated genome coverage). The results indicate that the success of high molecular weight DNA extractions is influenced by preservation methods. Although tested on nudibranchs, these findings are highly useful for genomic studies of other organisms, which may need to be preserved in remote locations before being transported to the laboratory for processing.

## Graphical abstract

This study aimed to identify the most suitable combination of four tissue preservation approaches and six DNA extraction methods to ensure high‐quality genomic DNA from nudibranchs, suitable for sequencing on the PacBio platform. Our study demonstrates that the success of DNA extractions is influenced by preservation methods, and highlights the necessity of balancing DNA yield, fragment size distribution, and availability of DNA for amplification. While tested on nudibranchs, these findings have great utility for genomic studies of other animals.

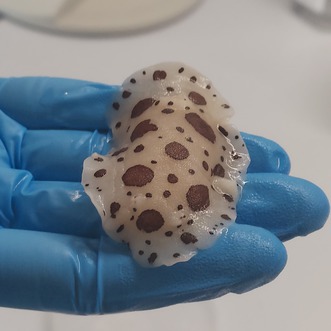

## 1 Introduction

High‐quality DNA extraction is ideal for genetic analyses. However, the quality requirements change over time depending on the purpose for which the DNA is used, generating the need for constant revisions of established protocols. In animal genetics, past scientific efforts focused on sequencing the cytochrome c oxidase subunit I (*cox1* gene, COI), a widely accepted marker for species identification (Folmer et al. 1994). COI sequencing has been instrumental in various applications, such as species identification, phylogenetic studies, and biodiversity assessments. Its usage remains popular in metabarcoding, a technique used to identify multiple species from a single environmental sample (Pentinsaari et al. 2016). Nevertheless, several studies have shown that whole genome sequencing, including shallow genome sequencing (genome skimming, 0.01‐5x coverage), is far superior for identifying novel animal species compared to COI sequencing (Straub et al. 2012; Trevisan et al. 2019; Marchán et al. 2020; Lou et al. 2021; Zhang et al. 2022). Whole genome sequencing on long‐read platforms, such as PacBio or Oxford Nanopore, is gaining popularity due to decreasing costs and advantages in genome assembly, particularly in resolving repetitive regions and complex genome structures. Consequently, methods for DNA extraction and sample preservation must be optimized to ensure that DNA fragment length is preserved to take full advantage of these sequencing technologies (Mayjonade et al. 2016).

The implementation of molecular tools for species identification, in combination with morphological data, is a keystone that is revolutionizing biodiversity research (Malinsky et al. 2018). Nudibranchs are one of the animal groups in which integrative taxonomy has impacted the most, resulting in the description of new intraspecific phenotypic plasticity and a wide variety of new cryptic species (Hirose et al. 2014; Hoover et al. 2015; Korshunova et al. 2019; Sørensen et al. 2020; Furfaro et al. 2022; Galià‐Camps et al. 2020; Dharmawan et al. 2021; Innabi et al. 2023; Pola et al. 2023; Ng et al. 2024). Although most of the nudibranch studies rely on a few markers, some taxa have not been fully resolved, even when using genome assembly drafts and mitogenome data (Galià‐Camps et al. 2024), evidencing that there is an urgent necessity for generating nudibranch reference genome assemblies for biodiversity assessment.

More than twenty years after the publication of the first mitochondrial genome of a nudibranch, *Roboastra europaea* (Grande et al. 2002), the first whole genome assembly of a nudibranch, *Berghia stephanieae*, has now become available (Goodheart et al. 2024). Whole genome sequencing of an additional 31 nudibranch species is currently in progress (https://goat.genomehubs.org/, consulted on 27th of February, 2025), which represents aorund ≈1% of the estimated 2545 nudibranch species worldwide (Gosliner et al. 2018; Sayers et al. 2024). Research interest in whole genome sequencing of nudibranchs is likely to grow in the coming years, as there is a need not only to resolve cryptic species differentiation from an evolutionary perspective, but also to uncover their unique chemical defense systems used to deter potential predators (Winters et al. 2021). Due to the complexity of molecular structures and the challenges of collecting sufficient animal samples for biochemical analysis, only about 10% of nudibranch species have been characterized for their bioactive molecules to date (Avila and Angulo‐Preckler 2020). The origin of most of these molecules is unclear; they might be synthesized directly by the animals, sequestered from toxin‐producing prey (Greenwood 2009), or produced by their symbiotic microbes (Džunková et al. 2023). Whole genome sequencing could reveal the origins and detailed structures of these compounds, potentially accelerating research with applications in the pharmaceutical industry.

Nevertheless, extracting DNA from nudibranchs is challenging due to their small size, thick, gluey mucus, and diverse pigments, which can interfere with DNA extraction, quantification, and sequencing. As shown in other mollusks, their mucus, composed of adhesive proteins, glycoproteins (mucins), and glycosaminoglycans (mucopolysaccharides), is often co‐extracted during nucleic acid purification, complicating downstream applications based on enzymatic reactions, such as DNA amplification and sequencing (Adema 2021; De Masi et al. 2015). Since nudibranchs are often collected in pristine areas far from molecular genetics laboratories, tissue preservation adds an additional level of difficulty to nudibranch genome sequencing, as it can negatively affect the quality of the extracted DNA (Wang et al. 2024). Maintaining nudibranchs in captivity for extended periods is nearly impossible due to their strict feeding habits (Gemballa and Schermutzki 2004), making the sacrifice of the animal after collection the most common practice. Therefore, in order to obtain genomic sequences of the highest quality, it is essential to identify the most suitable combination of tissue preservation and DNA extraction methods.

A recent study by Dahn et al. (2022) evaluated the effectiveness of a single high‐molecular‐weight DNA extraction protocol (Bionano Genomics) in combination with six different tissue preservation methods and six different tissue types from house mouse, zebra finch, two turtle species, American bullfrog, and zebrafish. However, it is widely known that DNA extraction protocols exhibit variable performance across different animal groups (Panova et al. 2016; Rodríguez et al. 2024). While there are well‐established protocols for DNA extraction from shell‐protected marine mollusks, such as bivalves (Sokolov 2000; Aranishi and Okimoto 2006; Pereira et al. 2011; Kurita and Kijima 2018), nudibranchs, being shell‐less, present unique challenges for DNA extraction. This is particularly due to the high amount of toxin‐containing mucus they produce and the presence of spicules in their mantle (Penney et al. 2020). Although there is a specialized PacBio Nanobind protocol for high‐molecular‐weight DNA extraction from *Aplysia*, a marine heterobranch mollusk with a small vestigial shell (ref: 102–580‐300 REV 02 DEC 2022), none of the currently available protocols for mollusks address the specific requirements for working with preserved tissue samples. It is important to note that most previous genetic studies on nudibranchs focused on sequencing the *cox1* gene and other molecular markers, which might not be useful for current long‐read sequencing efforts.

This study aims to identify the most effective combination of tissue preservation approaches and DNA extraction methods for long‐read sequencing of nudibranchs. Additionally, the quality of DNA extracted for PCR amplification of the *cox1* gene was assessed. Using a single nudibranch specimen, we compared four different preservation approaches (96% ethanol, drying, freezing, and 4°C storage as untreated control) in combination with six different DNA extraction protocols. While this work is the first to specifically focus on DNA extractions from nudibranchs, the insights gained regarding DNA sample preparation for long‐read whole genome sequencing are broadly applicable to other animal species.

## 2 Materials and Methods

### 2.1 Sampling and Species Identification

**FIGURE 1:**
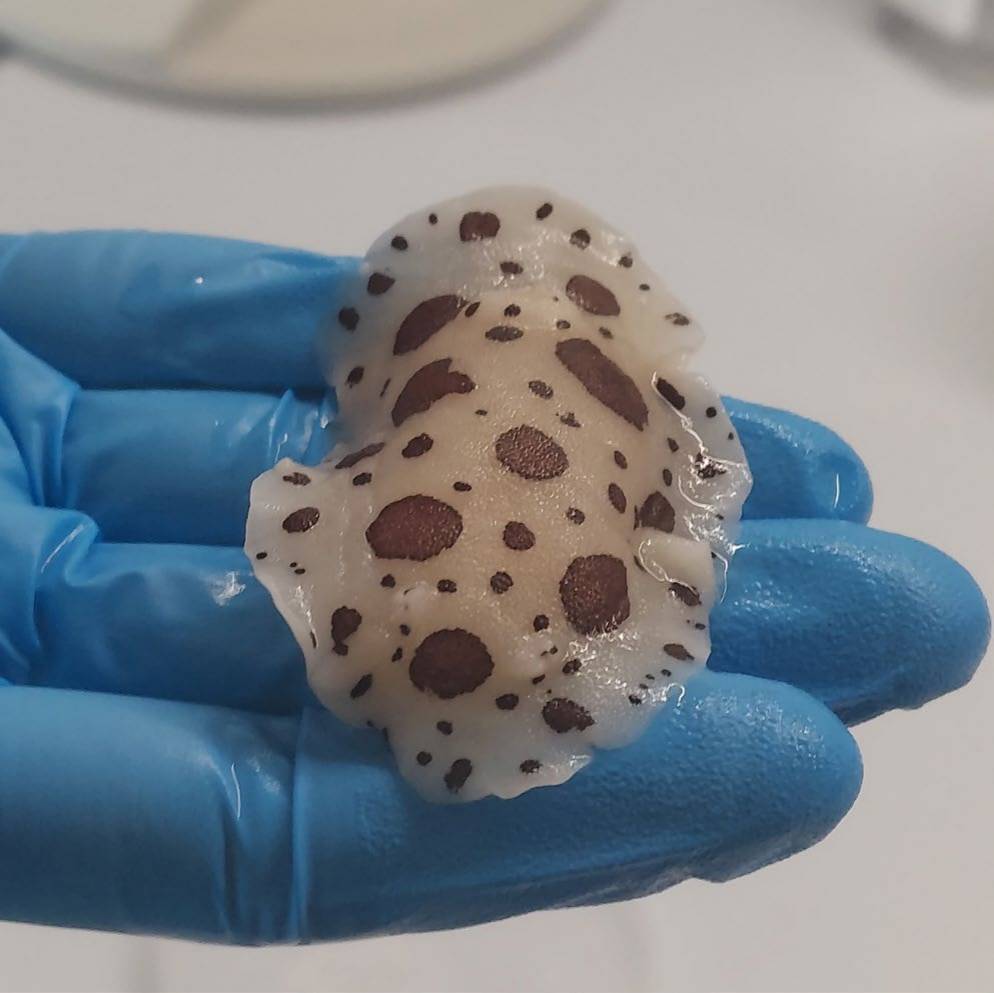
*Peltodoris atromaculata* specimen.

Two nudibranch specimens were collected by scuba‐diving in Cala Mateua (42°06′50.0″N, 3°10′01.8″ E), L'Escala, Catalonia (Spain) at 15 m deep on November 29th, 2023. They were identified in situ based on their phenotype as adults of *Peltodoris atromaculata* (Figure 1), commonly known as the dotted sea slug or sea cow, which is a dorid nudibranch with white pigmentation and brown patches, reaching up to 12 cm in length, found at depths of up to 50 m in the Mediterranean Sea (Thompson 1985) and some parts of the Atlantic, such as the Canary Islands (Perez et al. 1987). The specimens were shipped alive in sea water to the laboratory in Valencia (Spain), where they were sacrificed by separating the head from the body with a razor blade on November 30th, 2023. The stomach was dissected and removed in order to eliminate possible contaminating DNA, since this organ can be full of spicules from the sponges *Petrosia* or *Haliclona*, on which these animals feed (Gemballa and Schermutzki 2004). In addition, we also removed the excess slime which the animal produced in the laboratory. Each specimen was sliced with the help of a surgical blade into a total of 72 sub‐samples of an approximate weight of 30 mg and placed into 1.5 mL tubes.

The nudibranch specimens also underwent genetic analysis to corroborate the in situ identification of the species. The amplification of the *cox1* gene was performed according to Folmer et al. (1994), using 5 μL of 1 μM forward primer LCOI490: 5'‐GGT CAA CAA ATC ATA AAG ATA TTG G‐3' and 5 μL of 1 μM reverse primer HCO2198: 5'‐TAA ACT TCA GGG TGA CCA AAA AAT CA‐3' in a PCR reaction with KAPA HiFi HotStart ReadyMix 2x (Merck, Ref: KK5601). The reaction included 1 μL of DNA sample and 1.5 μL of water in a total reaction volume of 25 μL. The PCR program consisted of the initial denaturation at 95°C for 2 min and was followed by 30 cycles of 95°C for 30 s, 40°C for 1 min, and 72°C for 90 s, and the final extension step at 72°C for 2 min. The Sanger sequencing was conducted on the ABI 3730XL instrument.

### 2.2 Sample Preservation

The samples were preserved using three methods: (i) ethanol (E): samples were immersed in 96% ethanol and stored at 4°C until use; (ii) dried (D): samples were kept in the tube with the lid open for 3 days at room temperature (25°C) until dried, and afterwards, the tube was closed and stored at room temperature until use; (iii) frozen by nitrogen (N): samples were immersed in liquid nitrogen for 2 min and subsequently kept at −20°C until use. The fourth group of samples, which were stored at 4°C without any preservation method, was used as controls (untreated samples, U).

### 2.3 Initial DNA Extraction

Sub‐samples from the first nudibranch specimen were used for the initial DNA extraction using the following kits: BKL—Marine Animal Tissue Genomic DNA Extraction Kit (Bio Knowledge Lab, Ref: D2061); NEB—Monarch HMW DNA Extraction Kit (New England Biolabs, Ref: T3060L); PacBio—Nanobind Tissue Kit RT (PacBio, Ref: 102–302‐100); Qiagen—MagAttract HMW DNA Kit (Qiagen, Ref: 67563); and Promega—Wizard HMW DNA Extraction Kit (Promega, Ref: A2920). Additionally, we used a modified version of the custom protocol CTAB—cetyltrimethylammonium bromide DNA extraction protocol published by Chakraborty et al. (2020). All samples used in the initial preservation‐extraction combination testing came from the same nudibranch specimen to reduce variability, and each combination was tested in triplicate. This resulted in a total of 72 samples, comprising 24 preservation‐extraction combinations, each processed in triplicate.

For all DNA extraction protocols except for NEB, which employs its own tissue disruption method using provided pestles, samples were chopped with a surgical blade into pieces smaller than 1 mm in diameter and crushed in a Potter‐Elvehjem tissue homogenizer (Fisher Scientific, Ref: 15301331) in 1 mL of saline solution (0.9% NaCl). To minimize variability in sample size during homogenization, the three replicates were pooled together after homogenization, mixed, and then divided into three separate tubes. The cell homogenates were centrifuged at 5000 g for 1 min, and the supernatant was discarded.

The six protocols evaluated in this study employ different strategies for DNA extraction. All commercially available protocols (BKL, NEB, PacBio, Qiagen, Promega) were specifically developed to enrich high molecular weight DNA fragments. Protocol CTAB, on the other hand, has been used for DNA extractions before the commercial kit era and is still considered a gold standard for several applications in molecular biology (Schenk et al. 2023). Custom CTAB‐based protocols usually involve centrifugation for phenol/chloroform protein purification and ethanol/isopropanol precipitation of DNA at a relatively high speed, which, according to some studies, can reduce DNA fragment size (Kang et al. 2023). Nevertheless, high‐speed centrifugation is still employed to some extent in several commercial DNA extraction kits specifically designed for high molecular weight DNA. For example, in protocol BKL, spin columns with a silica gel solid base are used for DNA purification. While protocols NEB, PacBio, and Qiagen have replaced spin columns with kit‐specific alternatives, they still include steps with centrifugation speeds between 13,000 and 16,000 g. Protocol NEB is based on the attachment of long DNA fragments to specialized DNA Capture Beads with a 4 mm diameter made from borosilicate glass. In comparison, protocols PacBio and Qiagen recover DNA fragments using magnetic discs and beads, respectively. Interestingly, protocol Promega relies solely on DNA precipitation by gentle chemical agents and centrifugation, without using beads or columns, which somewhat resembles the non‐commercial protocol CTAB. Cut pipette tips were used in all protocols. Detailed protocols used in this study are available in the Supporting Information‐Methods.

After DNA extraction, samples were stored at −20°C. The DNA concentration was quantified on the Qubit dsDNA HS Assay Kit (Thermo Fisher, Ref: Q32854), using 1 μL of each sample, where the total extracted DNA amount of > 300 ng was considered sufficient for sequencing on the PacBio platform (Application note PN 101–995‐900 V2 NOV2020). In addition, 1 μL of each sample was used for DNA purity analysis on the NanoDrop 1000 Spectrophotometer (Thermo Fisher), where absorbance ratios A260/280 > 1.6 were considered suitable for long‐read sequencing.

The length of the extracted DNA fragments was analyzed in all samples using the 5300 Fragment Analyzer, for which highly concentrated samples were diluted to 5 ng/μL according to the manufacturer's instructions. The results were analyzed based on the fragment length of the peak with the highest DNA concentration (PHC). Preservation‐extraction combinations with a PHC of less than 7 kbp were excluded from further analysis. To assess the proportion of total DNA fragments in the sample represented by the PHC, we calculated the percentage of the total DNA concentration formed by the PHC, as indicated by the Fragment Analyzer. Samples were considered to have a uniform fragment size distribution if 50% of all fragments constituted the PHC.

### 2.4 COI Analysis by qPCR

The real‐time PCR (qPCR) of the *cox1* gene was performed with samples diluted to a concentration of 0.1 ng/μL. Preservation‐extraction combinations that did not reach the required concentration and fragment size were excluded from the qPCR analysis; however, to provide a complete overview of the qPCR performance of all extraction methods, we included at least one combination from each extraction protocol in the qPCR assay, even if they did not perform well in previous assays. Each sample was analyzed in triplicate, resulting in nine PCR replicates for each preservation‐extraction combination. As a positive control, five 1/10 serial dilutions of the Sanger‐sequenced sample were used. Additionally, a negative control containing water was included to detect primer‐dimers, which are non‐specific products from weak interactions between primers (Brownie et al. 1997). The detailed qPCR plate plan is available in Table S1.

The qPCR program consisted of the hot start at 95°C for 3 min, followed by 40 cycles of amplification at 95°C for 30 s, 40°C for 1 min, and 72°C for 90 s; and the melting curve step, which consisted of 1 cycle of 95°C for 30 s, 40°C for 30 s, and finally 95°C for 30s. The results were analyzed by comparing them to both positive and negative controls. The correct amplicon was expected to match the size of the positive control, without any primer dimers. The correct amplification was determined by comparing the proportion of fluorescence at the positive control peak position (Tm 80°C–82°C) with other peaks detected along the melting curve. If the Tm of the most prominent peak did not match the positive control position, the curve was categorized as deviated. For a sample to be classified as correctly amplified, at least two out of the three replicates had to meet these criteria.

### 2.5 Optimization of DNA Extractions

After analyzing the results for DNA yield, DNA fragment size, and qPCR, the most suitable preservation‐extraction combinations were selected for the following optimization phase, for which the preserved sub‐samples from the second nudibranch specimen were used. In this phase, modifications were made to the protocols of the commercial extraction kits aiming to obtain longer DNA fragments and higher concentrations, as described in Results.

### 2.6 DNA Sequencing

The preservation‐extraction combinations that yielded the most satisfactory results were selected for DNA sequencing. Samples were sheared using the Megaruptor 3 system (Diagenode, Belgium) at a speed of 35 to obtain an average fragment size of 10–12 Kbp. Libraries were prepared with the SMRTbell Prep Kit 3.0 (PacBio, USA) and, after verifying their concentrations and average sizes, pooled in equimolar amounts. Finally, sequencing primer annealing and polymerase binding were performed using the Sequel II Binding Kit 3.2 (PacBio, USA), followed by sequencing with the Sequel II Sequencing Kit 2.0 (PacBio, USA) on the Sequel II PacBio system. The initial sequence quality assessment was assessed by using fastp v0.24.0 (Chen et al. 2018).

## 3 Results

### 3.1 Total DNA Concentration and Purity

**FIGURE 2:**
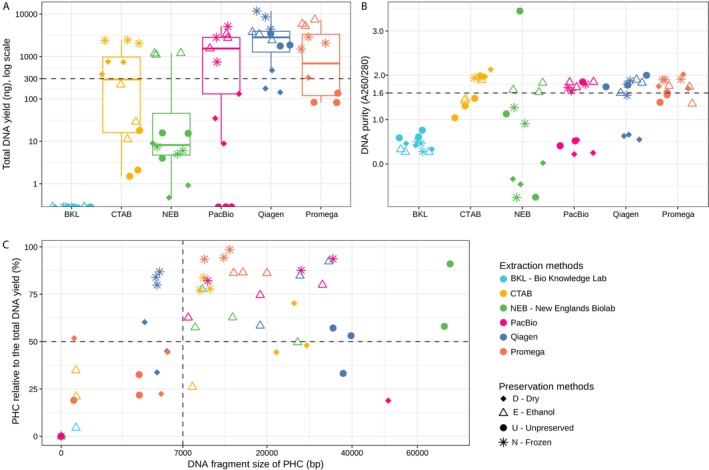
DNA extraction results of all preservation‐extraction combinations (A) Total DNA yield (ng) as measured by Qubit. The dashed line indicates the threshold of 300 ng. (B) Nanodrop DNA purity values A260/280, the dashed line indicates the threshold of 1.6. (C) Relation between fragment size of the peak with the highest DNA concentration (PHC) on the x‐axis and its proportion relative to the total DNA yield on the *y*‐axis, as indicated by the fragment analyzer. The dashed lines indicate the thresholds of 7000 bp for fragment size and 50% of the PHC proportion relative to the total DNA.

The DNA extraction results indicated that the total amount of extracted DNA depends not solely on the extraction protocol but also on its combination with the preservation method (Figure 2A, Table S2). As a consequence of the different preservation methods, the difference between the highest and lowest DNA amounts spanned more than 1 μg in all protocols except for protocol BKL; specifically, the ranges were 0.001–1.20 μg, 0.002–2.45 μg, 0–5.15 μg, 0.08–7.38 μg, and 0.143–11.80 μg for the NEB, CTAB, PacBio, Promega, and Qiagen methods, respectively. Protocol BKL was shown to be unsuitable for DNA extraction from nudibranchs, as it consistently produced 0 ng/μl, regardless of the preservation method used. Insufficient DNA amounts for PacBio sequencing (> 0.3 μg required) were also obtained using protocol NEB in all preservation methods (7.11 ± 5.45 ng), except for E, where it produced 1.16 ± 0.05 μg. Preservation methods D, E, and U significantly affected protocol CTAB, yielding as few as 0.32 ± 0.24 μg of total extracted DNA, while the combination of protocol CTAB with N preservation resulted in a ten‐fold increase to 2.30 ± 0.22 μg. A large effect of the preservation method was also observed in protocols PacBio and Promega. These protocols were negatively affected by preservation methods D and F, producing as little as 0–0.32 μg of DNA (average 0.08 ± 0.09 μg), whereas in combination with E and N preservation, they yielded up to 3.44 ± 2.03 μg. While protocols CTAB, NEB, PacBio, and Promega were negatively affected by two or three preservation methods, the performance of the protocol Qiagen decreased to 0.26 ± 0.18 μg only when combined with the D preservation, compared to 4.59 ± 0.26 μg with the other three preservation methods. Overall, the largest average DNA amounts were obtained from the Qiagen‐N combination (8.26 ± 3.70 μg), although this combination showed large variability across triplicates (4.42, 8.56 and 11.80 μg). Due to the consistently high yield and smaller variability across triplicates (5.24, 5.86 and 7.38 μg), the Promega‐E combination was also considered very efficient (Figure 2A, Table S2).

Taken together, the preservation methods with the largest average yields across all extraction protocols were E and N, with 2.19 ± 2.26 μg and 2.60 ± 3.22 μg, respectively. There was no significant difference between these two preservation methods (p‐value = 0.932, Wilcoxon rank‐sum test). The average values for E and N rise to 3.26 ± 2.03 μg and 3.90 ± 3.23 μg, respectively, when unsuitable combinations for PacBio sequencing (BKL‐E, CTAB‐E, BKL‐N, and NEB‐N) were discarded. In contrast, preservation methods D and U produced DNA yield sufficient for PacBio sequencing only when combined with the CTAB and Qiagen extraction protocols, respectively: 0.63 ± 0.21 μg for CTAB‐D and 2.36 ± 0.94 μg for Qiagen‐F. The yields obtained from the combinations involving preservation methods E and N were significantly higher than those with D and U (p‐value = 0.0003, Wilcoxon rank‐sum test).

As a consequence of low DNA yields, the sample purity expressed as A260/280 values resulting from extraction protocols BKL and NEB were 0.44 ± 0.16 and 0.79 ± 1.29, respectively, which are below the recommended threshold of 1.6 (Figure 2B). In contrast, while extraction protocol CTAB yielded low DNA amounts in combination with D, E, and U preservation methods, the resulting DNA purity was not negatively affected. In fact, protocols CTAB and Promega had the highest average A260/280 values: 1.71 ± 0.35 and 1.72 ± 0.21, respectively. More specifically, the combinations CTAB‐D and CTAB‐N had the highest A260/280 values, at 1.96 ± 0.02 and 2.03 ± 0.09, respectively. In terms of preservation methods, E yielded the highest average A260/280 values (1.46 ± 0.56), followed by N (1.37 ± 0.78), U (1.25 ± 0.87), and D, which did not yield DNA of sufficient purity in most combinations (0.82 ± 0.86).

Among the 24 preservation‐extraction combinations tested, only ten yielded both sufficient DNA amounts (3.16 ± 2.26 μg) and acceptable purity values (1.81 ± 1.16 A260/280): CTAB‐D, CTAB‐N, NEB‐E, PacBio‐E, PacBio‐N, Qiagen‐E, Qiagen‐F, Qiagen‐N, Promega‐E, and Promega‐N. To validate these results, we also assessed DNA purity by comparing Nanodrop and Qubit concentrations, where a Nanodrop/Qubit ratio close to 2 indicates high purity (Versmessen et al. 2024). Consistently, the ten samples with optimal A260/280 purity values also had Nanodrop/Qubit concentration ratios below 4 (Figure S1). Among them, samples processed with the Qiagen kit exhibited the highest purity (0.76 ± 0.33), followed by PacBio (3.23 ± 2.38). Preservations N and E yielded the lowest Nanodrop/Qubit concentration ratios (1.94 ± 1.24 and 2.58 ± 1.95, respectively) across all kits, except for NEB‐N and CTAB‐E, highlighting variation in sample purity across preservation‐extraction combinations.

### 3.2 DNA Fragment Size

The preservation‐extraction combinations were evaluated based on DNA fragment size, expressed as the PHC length and its concentration relative to the total extracted DNA, which reflects the long fragment extraction precision. Similar to the results of the total DNA yield analysis, fragment length was influenced by the preservation and extraction methods, making it impossible to identify a single method that guarantees sufficient fragment size independently of the combination in which it is used (Figure 2C, Table S2 and Figure S2). Of the ten combinations that performed well in both DNA yield and purity assessments (CTAB‐D, CTAB‐N, NEB‐E, PacBio‐E, PacBio‐N, Qiagen‐E, Qiagen‐F, Qiagen‐N, Promega‐E, and Promega‐N), eight also had sufficient PHC values (fragment length > 7 kb comprising ≥ 50% of the total DNA concentration). The exceptions were Qiagen‐N and CTAB‐D, which had limitations for long‐read sequencing: Qiagen‐N produced large DNA amounts (8.26 ± 3.70 μg) but with short fragment sizes (4.39 ± 0.18 kbp), while CTAB‐D yielded longer fragments (25.33 ± 3.30 kbp) but with a dispersed fragment size distribution, with only two of three replicates reaching the 50% PHC proportion, and it also produced one of the lowest DNA amounts (0.63 ± 0.21 μg).

Of the eight combinations that met the PHC thresholds, the average fragment length was 20.25 ± 10.69 kbp, comprising 75.56% ± 17.18% of the total DNA concentration (Figure 2C, Table S2). Among the four preservation methods analyzed, U yielded the longest fragment size (37.42 ± 2.40 kbp), but it was effective only when combined with the Qiagen extraction method. Preservation E achieved sufficient PHC length with four extraction protocols (NEB‐E, PacBio‐E, Qiagen‐E, Promega‐E), producing PHCs of 19.73 ± 8.60 kbp average size, while preservation N did so with three protocols (CTAB‐N, PacBio‐N, Promega‐N), yielding 15.23 ± 9.30 kbp PHCs, which indicates that E and N are the most suitable preservation methods to be combined with a variety of extraction protocols. In terms of extraction protocols analyzed across the eight combinations that met the PHC thresholds, the Qiagen extraction yielded PHCs with the largest average fragment size (31.97 ± 7.81 kbp), followed by PacBio (21.83 ± 11.47 kbp), NEB (16.27 ± 9.21 kbp), Promega (14.22 ± 3.45 kbp), and CTAB (9.72 ± 0.69 kbp).

Although the Qiagen‐U combination produced the longest fragment size (37.42 ± 2.40 kbp), its PHC comprised only 47.83% ± 12.81% of the total DNA concentration (with one of the three replicates failing to meet the 50% threshold), meaning that only about half of the extracted DNA fragments reached the longest fragment size (Figure 2C, Table S2). In comparison, although the combinations involving N preservation did yield PHCs of the longest fragment size (15.23 ± 9.30 kbp), they also comprised the largest proportion of the total extracted DNA (87.62% ± 7.79%). In summary, among the eight combinations that met the PHC thresholds, Promega was the best‐performing protocol in terms of PHC proportion of the total DNA extraction (90.89% ± 5.29%), and when combined with N preservation (Promega‐N), it produced the sharpest PHC, comprising 95.46% ± 2.68% of the total extracted DNA. Furthermore, the combination PacBio‐N also produced effective results as it was the third best protocol for PHC fragment size (24.11 ± 12.68 kbp) and second best for PHC proportion (87.80% ± 5.79%), which places it among the most suitable combinations for high molecular weight DNA extraction.

Of the remaining 14 preservation‐extraction combinations that did not yield sufficient DNA for sequencing, none produced fragment sizes meeting the PHC thresholds set in this study (Figure 2C, Table S2). In addition, although PHCs of 69.4 and 71.5 kb were observed in two out of three replicates of the NEB‐U combination, the low total DNA yield (11.73 ± 6.70 ng) makes it unsuitable for long‐read sequencing. Other combinations that produced long fragments, but with low DNA amounts and only in one of the three replicates, included BKL‐E, CTAB‐E, and PacBio‐D.

### 3.3 COI Analysis by qPCR

**FIGURE 3:**
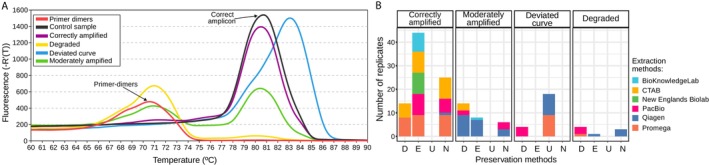
Results of the qPCR (A) Examples of melting curves. Control sample, in black, has a T_m_ of 80°C–82.5°C; and primer‐dimers control, in red, has a most prominent peak with a T_m_ of 70°C–72.5°C. The melting curves of the samples were divided into four types depending on their shape: Correctly amplified, moderately amplified, deviated curve, and a curve indicating a degraded sample. Details on each processed sample are available in Figure S3. (B) Bar diagram of qPCR results, where the kit‐preservation combinations are grouped according to the four types of melting curves obtained.

The melting curve analysis showed that the primer‐dimer peak and the peak corresponding to the amplicon of the control sample had Tm values of 70°C–72.5°C and 80°C–82.5°C, respectively. Using data on fluorescence and positions of the peaks across the melting curves, the samples were categorized into four groups: (i) correctly amplified—correct amplicon peak fluorescence > 700 and primer‐dimer peak fluorescence < 500, with the amplicon peak fluorescence being at least twice that of the primer‐dimer peak; (ii) degraded—fluorescence ratio between the amplicon and primer‐dimer peaks of < 0.6, making the primer‐dimer peak the most prominent one; (iii) deviated—the amplicon peak position did not match that of the control sample; (iv) moderately amplified—samples that did not meet the previous criteria, with a common example being an excessively high primer‐dimer peak compared to the amplicon peak (Figure 3A).

The average Cq (the cycle number at which the fluorescence signal of the amplified product crosses a predefined threshold) for correctly amplified samples was 24.00 ± 1.17 (Table S3), which matches the expected Cq of 23.92 corresponding to Sanger‐sequenced control samples with concentration 0.1 ng/μL. Samples with deviated curves presented a similar average Cq but with a large variability of 23.81 ± 3.33. For moderately amplified and degraded samples, the average Cq was 26.48 ± 1.10 and 27.75 ± 2.00, respectively, which indicates insufficient amplification of the DNA template.

In general, correct amplification was observed in all extraction methods combined with preservation E, except for Qiagen (Figures 3B and S3). In addition, extraction methods CTAB, PacBio, and Promega produced correct amplification when combined with preservation N, too. Dried samples (D) exhibited mixed results: combined with CTAB and Promega, extraction methods produced correctly amplified samples, while when combined with Qiagen, the extraction method resulted in moderate amplification. The satisfactory results of the D preservation in qPCR are in contrast to the insufficient DNA quality and DNA fragment length observed in previous analyses. Finally, the U preservation method was the least effective as it resulted in deviated amplification curves in all combinations.

From all extraction‐preservation combinations, the ones that yielded the best results in qPCR were CTAB‐E, CTAB‐N, NEB‐E, PacBio‐E, PacBio‐N, Promega‐E, and Promega‐N (Figures 3B and S3). In addition, BKL‐E also resulted in correct qPCR amplification; however, we do not recommend this method for high molecular weight extractions due to its low DNA yield (it was included in the qPCR experiment to test at least one sample from each extraction method). Similarly, the combination Promega‐D also produced correct amplification in the qPCR experiment, although it did not perform well in the parameters of DNA concentration and DNA fragment size. Notably, the Promega‐N combination was not only efficient in qPCR, but was also among the combinations that produced the longest DNA fragments. In contrast, the extraction method Qiagen produced only moderately amplified samples in the qPCR analysis, despite yielding long DNA fragments.

### 3.4 Optimization of DNA Extractions

**FIGURE 4:**
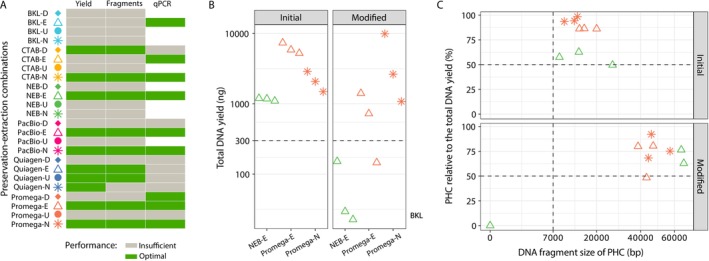
Comparison of the initial and modified DNA extractions (A). Summary of all results of all initial DNA extractions (B). Total DNA yield obtained from the initial DNA extractions NEB‐ethanol (NEB‐E), Promega‐ethanol (Promega‐E) and Promega‐frozen (Promega‐N), compared to their modified versions, as measured by Qubit. The dashed line indicates the threshold of 300 ng (C). Relation between fragment size of the peak with the highest DNA concentration (PHC) on the x‐axis and its proportion relative to the total DNA yield on the y‐axis, as indicated by the fragment analyzer, in the initial extractions compared to the modified versions of the three preservation‐extraction combinations as in panel B. The dashed lines indicate the thresholds of 7000 bp for fragment size and 50% of the PHC proportion relative to the total DNA.

To sum up results of all analyses, the best performing extraction‐preservation combinations were CTAB‐N, NEB‐E, PacBio‐E, PacBio‐N, Promega‐E, and Promega‐N, that yielded satisfactory DNA concentration and quality, DNA fragments of sufficient length, and they also produced correct qPCR amplification of the *cox1* gene (Figure 4A). In the subsequent phase, our goal was to modify extraction protocols of commercially available kits to achieve higher DNA concentrations and increased DNA fragment size. However, the protocols of these kits offer only limited options for further improvements. We made slight modifications in the protocols NEB and Promega. In the extraction protocol NEB, the samples were not crushed with the pestle from the kit, but rather processed with the Potter Elvehjem tissue homogenizer (Fisher Scientific, Ref: 15301331). In protocol Promega, the samples were incubated with the lysis buffer (step 3 of protocol Promega, Supporting Information) for 30 min, instead of 15 min previously used.

Surprisingly, in the two extraction methods combined with the E preservation method (Promega‐E and NEB‐E), a significant decrease in DNA concentrations was observed (p‐value = 0.015, Wilcoxon rank‐sum test) in comparison with the same combinations from the initial extractions set, representing an average 8.8‐fold reduction (Figure 4B, Table S2). In contrast, the only combination that did not involve the E preservation method, Promega‐N, showed no reduction in DNA yield. In fact, one of the triplicates exhibited a 5‐fold increase in DNA yield compared to the initial extraction average.

Although protocol modifications did not increase DNA yields, they resulted in a 4.6‐fold increase in DNA fragment length across the three preservation‐extraction combinations tested in the second assay (Figures 4C and S2). The combinations NEB‐E, Promega‐E, and Promega‐N increased median DNA fragment length from 13.9, 15.6, and 12.5 kb to 64.4, 43.1, and 45.8 kbp, respectively, demonstrating a positive outcome of the protocol modifications.

### 3.5 DNA Sequencing

**FIGURE 5:**
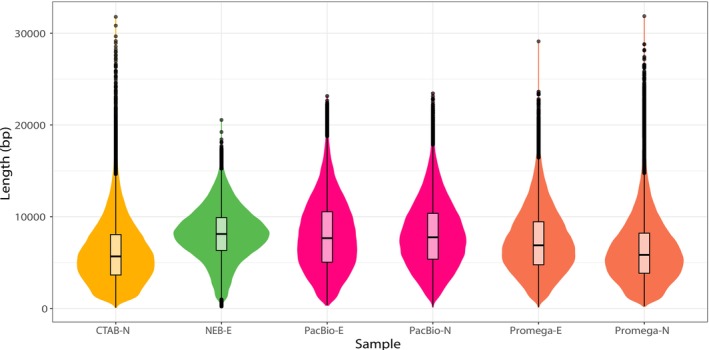
Read lengths obtained from the DNA sequencing. Read length distributions obtained from the six DNA samples that were sequenced on the PacBio platform. Box plots indicate medians, and colors denote extraction methods used.

The sequencing on the PacBio platform yielded satisfactory results, with an average of 83,150 ± 10,111 reads per sample and an average read length of 7297 ± 873 bp, resulting in a total of 3.6 Gbp, equivalent to 3.2× the estimated genome size of nudibranchs (Figure 5). The longest fragment sizes (after DNA shearing with the Megaruptor) were observed in sample NEB‐E (8069.43 ± 2836.45 bp, median 8111 bp), followed by PacBio‐N (8018.72 ± 3571.60 bp, median 7753 bp) and PacBio‐E (7945.45 ± 3806.27 bp, median 7661 bp). The GC content (38.2%) remained consistent across all six samples.

## 4 Discussion

The results of our study demonstrate that both the choice of extraction protocol and preservation method play a critical role in determining the success of DNA extraction for long‐read sequencing applications. While certain combinations yielded high DNA concentrations, others were more efficient in preserving long DNA fragments. This is the first study in animals that, in addition to evaluating different tissue preservation approaches combined with various DNA extraction methods, focuses on the three key aspects essential for successful long‐read sequencing: DNA concentration, DNA fragment length, and DNA purity.

The untreated samples stored at 4°C (U) produced the worst results across all three evaluated aspects, likely due to tissue degradation caused by enzymatic breakdown and activity of psychrophilic microbes, as reported in previous studies on unpreserved samples (Zhang et al. 2019; Pérez‐Brocal et al. 2020). Bacterial and fungal contamination may result in an apparently high DNA yield; however, this contamination can be indicated by large Cq variability and primer dimers in qPCR, as not all extracted DNA belongs to the target animal species. Additionally, in the case of nudibranchs, their mucus may interfere with DNA extraction reagents (Ayyagari et al. 2017; Chakraborty et al. 2020). Since most of the nudibranch samples cannot be processed immediately after sampling, the selection of the most suitable preservation method is essential for the subsequent DNA extractions. Our results showed that air drying (D), despite being known to reduce mucus viscosity and its structural integrity (Webster and Tarran 2018), also leads to unsatisfactory DNA extraction results. DNA in air‐dried samples was probably compromised by oxidative stress and ongoing enzymatic breakdown which continues actively during drying at room temperature, resulting in insufficient DNA yields and short fragment sizes. However, these effects seem to depend on the resistance of the animal cells; for instance, a previous study on a porcelaneous foraminifera (single‐celled Rhizarian protists) or flies from the genus *Simulium* did not report any negative effects of air drying on DNA extraction (Lyu et al. 2018; Post et al. 1993), and DNA extractions from dry skin samples of museum specimens have also been reported (Burrell et al. 2015).

In contrast, freezing samples (N) or preserving them in ethanol (E) yielded satisfactory results in our study, though only when paired with the appropriate extraction method. The high variability across preservation‐extraction combinations might explain the discrepancies in previous reports. Residual ethanol is known to inhibit PCR (Demeke and Jenkins 2010) and is also believed to interfere with DNA extraction reagents (Johnson et al. 2023). Nevertheless, several studies have reported no negative effect of ethanol preservation on DNA extractions (Palero et al. 2010; Bressan et al. 2014; Johnson et al. 2023), although only one study focused specifically on high molecular weight extractions for long‐read sequencing (Dahn et al. 2022). In our study, no negative effects of ethanol (E) on DNA fragment size were observed. However, results from the modification stage (second assay) indicated that extended ethanol preservation might reduce DNA yields. While slight modifications to the original protocols in our study increased the DNA fragment sizes, DNA yields were likely impacted by prolonged ethanol preservation (3 months), even at −20°C. This aligns with prior findings, which showed that short‐term ethanol preservation (1 week) does not affect DNA fragment size (Dahn et al. 2022), but extended preservation (years) may hinder DNA extractions (Barnes et al. 2000; Precioso et al. 2022).

Similarly, the impact of sample freezing (N) on DNA extraction has been debated. Freezing can improve DNA yields by enhancing cell lysis—the first step of DNA extraction (Armbrecht et al. 2020); however, some researchers avoid freezing samples to prevent ice crystal formation, which can affect DNA fragment size, or to avoid thawing that could activate nucleases and degrade the DNA (Trigodet et al. 2022). In our study, combinations of the Promega (Wizard), PacBio (Nanobind), and NEB (Monarch) extraction kits applied to ethanol‐preserved (Promega‐E, PacBio‐E, NEB‐E) or frozen samples (Promega‐N, PacBio‐N) proved to be among the most efficient in terms of DNA concentration, fragment length, and amplification. This is consistent with a prior study testing different preservation methods alongside a single high molecular weight DNA extraction kit (Dahn et al. 2022).

It is important to emphasize the efficiency of the custom CTAB extraction protocol, particularly when applied to frozen samples (CTAB‐N). Although numerous commercial kits are available for high molecular weight DNA extractions, custom protocols that employ CTAB for cell lysis, phenol‐chloroform purification, and ethanol/isopropanol precipitation remain widely used, despite their lower reproducibility and the toxicity risk CTAB and phenol‐chloroform pose to the user (Isomaa et al. 1976; Gami et al. 2014). In fact, the DNA of the nudibranch *Berghia stephanieae*, used for the first nudibranch genome sequencing, was extracted using a custom CTAB‐based protocol (Goodheart et al. 2024). The custom protocols offer several advantages, including lower cost and customization, which is especially important in regions where commercial kits are not readily available. The custom CTAB‐based protocol is often preferred in laboratories that do not process large numbers of samples, where protocol customization for difficult‐to‐process samples is prioritized over reproducibility. In summary, for high molecular weight extractions, laboratories preferring commercial solutions may opt for Promega (Wizard), PacBio (Nanobind), and NEB (Monarch) kits, though the custom CTAB‐based protocol remains a strong alternative.

It is well‐known that DNA molecules can be fragmented by pressure. In fact, high pressure, such as that used in hydroshearing or sonication, is commonly employed in procedures where DNA fragments of a specific size are required (Sun et al. 2022). For this reason, several commercial kits specifically designed for high molecular weight DNA use alternative approaches, such as magnetic beads, instead of centrifugation (Jones et al. 2021). Interestingly, contrary to common belief, only one study reported a decrease in DNA fragment size caused by centrifugation using common benchtop equipment (Kang et al. 2023). In our study, the most effective extraction methods, Promega and CTAB, involve centrifugation at 16,000 × g for 2 min or 13,000 × g for 10 min, suggesting that these centrifugation conditions do not adversely affect DNA fragment size and are compatible with long‐read sequencing. Attempting to maximize fragment length is often counterproductive, as it typically results in lower DNA yields, which can negatively impact sequencing performance, particularly on the Oxford Nanopore platform (De La Cerda et al. 2023). For PacBio sequencing, achieving extremely long fragments is also unnecessary, as the library preparation process includes Megaruptor DNA shearing to standardize fragment length to approximately 10 kbp.

We assessed DNA yield, fragment length, and DNA purity to thoroughly evaluate the suitability of preservation‐extraction combinations for long‐read whole genome sequencing. While the extraction optimization studies from the Sanger sequencing era used to focus only on *cox1* gene amplification, our findings suggest that preservation‐extraction combinations effective for *cox1* gene amplification may not meet the DNA yield and fragment size requirements for long‐read whole genome sequencing. We emphasize the need to carefully balance DNA concentration, DNA fragment length, and DNA purity. The PacBio platform employs real‐time sequencing with uninterrupted template‐directed synthesis by DNA polymerase; therefore, DNA accessibility for amplification, for example, qPCR amplification of the *cox1* gene, is an additional way of assessing DNA purity before conducting long‐read whole genome sequencing.

In conclusion, we demonstrated that the effectiveness of high molecular weight DNA extraction protocols varies depending on the preservation method used. Among the commercial protocols, the combination of Wizard (Promega) and Nanobind (PacBio) kits for both frozen and ethanol‐preserved samples, and the ethanol‐preservation paired with Monarch (NEB) kits produced the most favorable results. Additionally, the custom CTAB‐based protocol remains a highly suitable option due to its greater flexibility, which is an advantage when working with nudibranch or other organisms. Extraction protocols applied to frozen samples had the best performance, although ethanol preservation could be used as an alternative in most cases. All six methods (CTAB‐N, NEB‐E, PacBio‐E, PacBio‐N, Promega‐E, and Promega‐N) yielded satisfactory results in our pilot PacBio sequencing run, though additional replicates across different sequencing runs and instruments are needed to further support our findings.

## Author Contributions

**Inés Alberola‐Mora:** investigation (equal), methodology (equal), project administration (equal), validation (equal), visualization (equal), writing – original draft (equal), writing – review and editing (equal). **Oleanna Guerra‐Font:** investigation (equal), methodology (equal), validation (equal), visualization (equal), writing – original draft (equal), writing – review and editing (equal). **Omar Daniel Espinoza‐Calderón:** methodology (equal), project administration (equal), writing – review and editing (equal). **Carles Galià‐Camps:** conceptualization (equal), funding acquisition (equal), project administration (equal), resources (equal), writing – original draft (equal), writing – review and editing (equal). **Mária Džunková:** conceptualization (equal), data curation (equal), formal analysis (equal), funding acquisition (equal), investigation (equal), methodology (equal), project administration (equal), resources (equal), supervision (equal), validation (equal), visualization (equal), writing – original draft (equal), writing – review and editing (equal).

## Conflicts of Interest

The authors declare no conflicts of interest.

## Supporting information


Data S1.

Figure S1.

Figure S2.

Figure S3.

Tables S1–S3.

## Data Availability

All data are included in the manuscript and are publicly available. The sequencing data are available at NCBI SRA with BioProject ID: PRJNA1230873.
