# Supplementary material for: Combination of Sample Preservation Approaches and DNA Extraction Methods for Long‐Read Sequencing of Nudibranchs' Genomes
Source: Ecol Evol. 2025 Apr 15;15(4):e71262. doi: 10.1002/ece3.71262 (PMC11997370; doi:10.1002/ece3.71262)
Supplement: Supplementary file 1 — Data S1. [file ECE3-15-e71262-s003.pdf]

Supporting Information

Methods

Index

BKL - Marine Animal Tissue Genomic DNA Extraction Kit (Bio Knowledge Lab, Ref: D2061) 2
CTAB - Cetyltrimethylammonium bromide (CTAB) protocol (Chakraborty et al., 2020).................... 3
NEB - Monarch® HMW DNA Extraction Kit (New England Biolabs, Ref: T3060L) 5
PacBio - Nanobind® tissue kit RT (PacBio, Ref: 102-302-100) Extracting HMW DNA from Aplysia tissue using Nanobind® kits 7
Qiagen - Qiagen MagAttract HMW DNA Kit (REF: 67563) 9
Promega - Wizard HMW DNA Extraction Kit (Promega, REF: A2920).. 10

BKL - Marine Animal Tissue Genomic DNA Extraction Kit (Bio Knowledge Lab, Ref: D2061)

1. Process sample as described in **MATERIALS AND METHODS**. The protocol starts with the sample centrifuged after homogenization.

2. Add 500 µL MA Tissue-1 Buffer and mix using 1,000 µL wide bore pipette tips.

3. Add 40 µL Proteinase K (20 mg/mL). Mix well.

4. Incubate at 65 ºC for 60 min. Mix 2-3 times by inversion during incubation.

5. Centrifuge at 13,000 g for 5 minutes. Transfer the supernatant to a fresh tube.

6. Add 20 µL RNASE-A 20 µg/µL and incubate for 5 minutes at room temperature.

7. Add 400 µL MA Tissue-2 Buffer and mix by inversion.

8. Transfer up 700 µL mixture to a High-Q™ Spin Column placed into a Collection Tube.

9. Centrifuge at 10,000 g for 1 minute. Remove the flow-through and place back the High-Q™ Spin Column into a Collection Tube. Repeat steps 8 and 9 with the remaining mixture.

10. Add 500 µL WB2 Buffer.
Check absolute ethanol has been added to WB2 Buffer.

11. Centrifuge at 10,000 g for 30 seconds. Discard the flow-through and place back the High-Q™ Spin Column into a Collection Tube. Repeat steps 10 and 11 one more time.

12. To dry the High-Q™ Spin Column and eliminate residual ethanol, centrifuge again at 13,000 g for 1 minute.

13. Place the High-Q™ Spin Column into a clean 1.5 mL Tube. Add 100 µL prewarmed Elution Buffer or Water (Molecular Biology Grade).

14. Incubate at room temperature for 2 minutes. Centrifuge at 13,000 g for 1 minute to elute purified DNA.

CTAB - Cetyltrimethylammonium bromide (CTAB) protocol (Chakraborty et al., 2020)

Prepare 1M Tris-HCl
• Tris Base 121.1 g
• HCl Make pH 8.0
• Distilled Water Adjust volume to 1 L

Prepare 0.5 M EDTA
• disodium dihydrate EDTA disodium dihydrate 186.1 g
• NaOH - Make pH 8.0
• Distilled Water - Adjust volume to 1 L

Prepare 5 M Sodium chloride
• NaCl 292.2 g
• Distilled Water Adjust volume to 1L

Prepare TE Buffer
• 1 M Tris-HCl 0.5 mL
• 0.5 M EDTA 100 µL
• Distilled Water to 50 mL

Prepare CTAB-Lysis Buffer
• Tris–HCl (pH 8.0) 100 mM
• NaCl 1.4 M
• EDTA disodium dihydrate 20 mM
• Hexadecyltrimethylammonium bromide (CTAB) 2% w/v
• Polyvinyl pyrrolidone 2% w/v
• β-Mercaptoethanol 0.2% v/v

Prepare 3 M Sodium Acetate
• Sodium acetate trihydrate 102.025 g
• Glacial acetic acid Adjust pH to 5.2
• Distilled Water Adjust volume to 250 mL

1. Process sample as described in **MATERIALS AND METHODS**. The protocol starts with the sample centrifuged after homogenization.

2. Resuspend the sample in 400 µL of salt solution (0.9% NaCl), mix using 1,000 µL wide bore pipette tips. Add 400 µL of CTAB-lysis buffer pre-heated to 60 °C. Vortex briefly.

3. Add 20 µL Qiagen® Proteinase K and two 2.8 mm metallic beads (Merck, Ref: BMSD113328), incubate in the thermoblock for 1 hour at 60 °C with 300 rpm shaking.

4. Add 820 µL of chloroform: isoamyl alcohol (24:1), vortex for 1 min and centrifuge at 12,000 rpm for 5 min at 4 °C.

5. Collect approx. 700 µL of supernatant above the white layer and place it into a new 2 mL tube. Add 700 µL of chloroform: isoamyl alcohol (24:1), vortex for 1 min and centrifuge at 12,000 rpm for 5 min at 4 °C.

6. Collect approx. 600 µL of supernatant above the white layer and place it into a 1.5 mL tube, in which you already have 66 µL 3 M Sodium (so its final concentration will be 0.3 M). Mix by inverting the tube several times.

7. Add 466 µL of isopropanol (which is 0.7 x volume of the sample) and shake a lot to see bubbles.

8. Incubate for 10 min at room temperature, vortex again and then store for 18 hours at −80ºC.

9. Centrifuge 45 min at 4 ºC at 13,000 g.

10. Remove supernatant. Add 500 µL of 70 % ethanol. Vortex and centrifuge at 13,000 g for 10 min at 4 ºC.

11. Discard the supernatant and centrifuge shortly again to collect and discard all remaining ethanol. Let remaining ethanol dry out by brief incubation in a thermoblock set to 37 ºC.

12. Resuspended in 30 µL water.

NEB - Monarch® HMW DNA Extraction Kit (New England Biolabs, Ref: T3060L)

Part 1: TISSUE LYSIS

1. Following the protocol for low input, prepare the lysis mastermix by combining 300 µL of HMW gDNA Tissue Lysis Buffer with 10 µL of Proteinase K per sample.

2. Transfer tissue to a Monarch Pestle Tube. Place on ice (frozen samples). Dry, fresh and ethanol samples are placed at room temperature.

3. Homogenization:
   a. Use the pestle to grind the sample within the pestle tube; leave the pestle in the tube.
   b. Using a wide bore pipette tip, add 300 µL of the lysis master mix to the sample. Do not dispose of this tip yet, as it will be used to mix the sample.
   c. Ensure there is no tissue material remaining on the pestle, then discard the pestle. If tissue material sticks to the pestle, transfer it carefully into the tube.
   d. Using the wide-bore tip, pipette up and down a few times to homogenize the tissue lysate to ensure rapid, complete lysis. Discard the pipette tip.

4. Incubate at 56 °C for 45 minutes in a thermal mixer with 500 rpm agitation.

5. Add 5 µL of RNase A and mix by inverting 10 times. Incubate for 10 minutes at 56 °C at 500 rpm.

6. Change the heat block in the thermal mixer to accommodate a 2 mL tube, and preheat the block to 56 °C.

7. Add 150 µL of Protein Separation Solution and mix by inverting for 1 minute using a vertical rotating mixer at 20 rpm.

8. Centrifuge for 10 minutes at 16,000 x g.

9. If working with multiple samples, prepare and label the plastics for the upcoming steps. Each sample will require (1) Monarch Collection Tube II, (1) Monarch Bead Retainer, (2) Monarch 2 mL Tubes, (1) LoBind DNA tube (Eppendorf, Ref: 30108051).

10. Using a 200 µL wide-bore pipette tip, transfer the upper phase containing the DNA (large, clear phase) to a labeled Monarch 2 mL Tube. Highest yields will be achieved by transferring as much of the upper phase as possible. Using a 200 µL wide-bore pipette tip to transfer the final volume of upper phase is recommended for maximum yield.
    - Avoid transferring material from the protein layer, although a small amount (1-2 µL) will not be detrimental.
    - If protein enters the pipette tip, gently push it back into the tube.
    - If a lower protein phase is not visible, leave ~30 µL behind to ensure protein is not carried over.

Part 2: HMW gDNA BINDING AND ELUTION

1. Using clean forceps, add 2 µL of DNA Capture Beads to each sample, which should be contained in a Monarch 2 mL Tube.

2. Add 275 µL isopropanol, close the cap, and mix on a vertical rotating mixer at 10 rpm for 5 minutes to attach DNA to the beads.

3. Remove liquid by pipetting. Avoid removing any of the gDNA wrapped around the glass beads. For optimal DNA solubility, avoid letting the bound DNA dry out on the beads during this and the following steps; add the next buffer quickly. Keeping the tube upright, insert pipette tip and gently push beads aside to remove liquid, or tilt the tube almost horizontally and remove liquid from the top of the angled tube.

4. Add 500 µL gDNA Wash Buffer, close the cap and mix by inverting the tube 2-3 times. Remove the wash buffer as described in the previous step.

5. Repeat the wash in Step 4, and remove the wash buffer by pipetting.

6. Place a labeled bead retainer into a Monarch Collection Tube II. Pour the beads into the bead retainer and close the cap. Discard the used Monarch 2 mL Tube.

7. Pulse spin (≤ 1 second) the sample in a benchtop minicentrifuge to remove residual wash buffer from the beads.

8. Separate the bead retainer from the collection tube, pour the beads into a new, labeled Monarch 2 mL Tube, and insert the used bead retainer into a labeled 1.5 mL LoBind tube. Discard the used collection tube.

9. Immediately add 100 µL Elution Buffer II onto the glass beads and incubate for 5 minutes at 56 °C in a thermal mixer with agitation at 300 rpm. Halfway through the incubation, ensure beads are not stuck by tilting the tube almost horizontally and gently shaking; do not let liquid reach the cap and avoid splashing.

10. Ensure bead retainer is inserted into the 1.5 mL microfuge tube. Pour eluate and beads into the bead retainer and close the cap.

11. Centrifuge for 30 seconds at 12,000 x g to separate the eluate from the glass beads. Discard the beads and retainer.

12. Pipette the eluate up and down 5-10 times with a wide bore pipette tip and ensure any visible DNA aggregates are dispersed. Samples can be stored at 4 °C for short term use (weeks) or -20 °C for long term storage.

PacBio - Nanobind® tissue kit RT (PacBio, Ref: 102-302-100) Extracting HMW DNA from *Aplysia* tissue using Nanobind® kits

1. Process sample as described in **MATERIALS AND METHODS**. The protocol starts with the sample centrifuged after homogenization. Keep the tube on ice during the entire disruption process.

2. Add 750 µL of cold Buffer CT. mix using 1,000 µL wide bore pipette tips. Buffer CT should be kept on ice when removed from the refrigerator.

3. Transfer homogenate and any foam to a 2 mL Protein LoBind microcentrifuge tube. The 2 mL tube is essential for efficient lysis because of its shape; the narrow taper of a 1.5 mL tube prevents proper mixing of the lysate during thermomixing.

4. Pellet homogenate by centrifuging at 1,500 x g at 4°C for 5 min. Discard supernatant.

5. Add 1 mL of cold Buffer CT and pipette mix 10X with a wide bore P200 pipette to resuspend tissue.

6. Pellet homogenate by centrifuging at 1,500 x g and 4 °C for 5 min. Discard supernatant.

7. Pulse vortex pellet 1 s x 2 times (max setting) to dislodge pellet.

8. Add 20 µL of Proteinase K to the previous pellet. Add 150 µL of Buffer CLE3 and pipette mix 10X with a wide bore P200 pipette.

9. Incubate on a ThermoMixer at 55 °C and 900 rpm for 30 min. Spin the tube on a mini-centrifuge for 2 s to remove liquid from the cap.

10. Add 20 µL of RNaseA.

11. Repeat step 10.

12. Add 60 µL of Buffer SB and pulse vortex for 1 s x 5 times (max setting) to mix.

13. Centrifuge at 10,000 x g and RT (15–30°C) for 5 min.

14. Transfer up to 300 µL of supernatant to a new 1.5 mL Protein LoBind microcentrifuge tube using a wide bore P200 pipette. Discard the 2 mL Protein LoBind microcentrifuge tube containing the precipitated pellet.

15. Add 50 µL of Buffer BL3 to the previous supernatant and inversion mix 10X.

16. Spin the tube on a mini-centrifuge for 2 s to remove liquid from the cap.

17. Add Nanobind disk to lysate and add 350 µL of isopropanol. Inversion mix 10X. The Nanobind disk must be added before isopropanol. A large, cloudy mass appeared upon addition of isopropanol and inversion mixing; this adhered to the Nanobind disk and became clear during the next step.

18. Mix on a platform rocker at 20 rpm for 15 min at RT.

19. Place the tube rack on the magnetic base.

20. Discard the supernatant with a pipette, taking care to avoid pipetting the DNA or contacting the Nanobind disk.

21. Add 500 µL of Buffer CW1, remove tube rack from magnetic base, inversion mix 4X, replace the tube rack on the magnetic base, and discard the supernatant.

22. Repeat step 21.

23. Add 500 µL of Buffer CW2, inversion mix 4X, replace the tube rack on the magnetic base, and discard the supernatant.

24. Repeat step 23.

25. Pipette out any residual liquid from the tube cap.

26. Spin the tube on a mini-centrifuge for 2 s. With the tube rack already on the magnetic base and right-side-up, place the tube rack and remove residual liquid. If the Nanobind disk is blocking the bottom of the tube, gently push it aside with the tip of the pipette towards the magnet.

27. Repeat step 26.

28. Add 75 µL of Buffer EB directly onto the Nanobind disk and incubate at RT for 10 min. The Nanobind disk does not need to be fully immersed in Buffer EB – it need only be wetted and sitting atop the liquid.

29. Collect DNA by transferring eluate to a new 1.5 mL microcentrifuge tube using a wide bore P200 pipette. DNA LoBind tubes can be used in this step.

30. Spin the tube containing the Nanobind disk on a mini-centrifuge for 5 s. Use a standard P200 pipette to combine any additional liquid that comes off the disk with the previous eluate. Repeat if necessary.

31. Pipette mix 5X with a standard P200 pipette to homogenize the eluate and disrupt any unsolubilized "jellies" that may be present. Limited pipette mixing will not noticeably reduce DNA size or sequencing read lengths but is important for accurate quantitation and consistent sequencing performance. Take care to disrupt any regions that feel more viscous than other regions.

32. Let eluate rest overnight at RT to allow DNA to solubilize. Visible "jellies" should disperse after resting. The extracted HMW DNA can be heterogeneous. This is normal and is one of the challenges of working with HMW DNA. The bigger the DNA, the more this will be apparent.

Qiagen - Qiagen MagAttract HMW DNA Kit (REF: 67563)
Quick-Start Protocol

1. Process sample as described in **MATERIALS AND METHODS**. The protocol starts with the sample centrifuged after homogenization.

2. Resuspend the sample in 200 µL of salt solution (0.9% NaCl), mix using 1,000 µL wide bore pipette tips. Add 20 µL Proteinase K.

3. Add 4 µL RNase A solution and 150 µL Buffer AL to the sample. Mix carefully by pulse-vortexing. Do not add Proteinase K directly to Buffer AL.

4. Incubate at room temperature for 30 min. Briefly centrifuge the 2 mL microcentrifuge tube to remove drops of liquid from inside the lid.

5. Add 15 µL MagAttract Suspension G to the sample. Add 280 µL Buffer MB to sample. Place microcentrifuge tube with sample into tube holder.

6. Place tube holder onto mixer. Incubate at room temperature for 3 min at 1,400 rpm.

7. Place the tube holder on the magnetic base, wait until bead separation has been completed (~1 min), and remove the supernatant.

8. Add 700 µL Buffer MW1 to the sample and place the tube holder onto the mixer. Incubate at room temperature for 1 min at 1,400 rpm.

9. Place the tube holder on the magnetic base, wait until bead separation has been completed (~1 min), and remove the supernatant.

10. Repeat step 9..

11. Add 700 µL Buffer PE to the sample and place the tube holder onto the mixer. Incubate at room temperature for 1 min at 1400 rpm.

12. Place the tube holder on the magnetic base, wait until bead separation has been completed (~1 min), and remove the supernatant.

13. Repeat steps 11. Use a small pipette tip to remove all traces of Buffer PE.

14. Rinse the particles with 700 µL distilled water while the beads are fixed to the walls of the microcentrifuge tube. Incubate for 1 min and remove the supernatant.
    Important: Pipet water into microcentrifuge tube against the side away from the bead pellet. All pipetting steps must be done carefully to avoid disturbing the fixed bead pellet.

15. Repeat step 13.

16. Remove the tube holder from the magnetic base and add 100 µL Buffer AE. Incubate at room temperature for 3 min at 1,400 rpm.

17. Place tube holder onto magnetic base, wait until bead separation is completed (~1 min), and transfer supernatant with high-molecular-weight DNA to a new microcentrifuge tube.

Promega - Wizard HMW DNA Extraction Kit (Promega, REF: A2920)

Isolating HMW Genomic DNA from Plant Tissue

1. Process sample as described in **MATERIALS AND METHODS**. The protocol starts with the sample centrifuged after homogenization.

2. Add 500 µL of HMW Lysis Buffer A, and vortex 1–5 seconds to wet the tissue.

3. Incubate at 65 °C for 15-30 minutes (30 min is recommended).

4. Cool the lysate to room temperature for 5 minutes. Add 3 µL of RNase A Solution to the sample and mix by inverting the tube 5–7 times. Incubate the mixture at 37 °C for 15 minutes.

5. Add 20 µL of Proteinase K Solution to each leaf sample and mix the sample by inverting the tube 10 times. Incubate the mixture at 56 °C for 15 minutes. Cool to room temperature for at least 5 minutes or chill on ice for 1 minute.

6. Centrifuge at 13,000 × g for 3 minutes at room temperature to pellet any insoluble material. Transfer the lysate to a clean 1.5 mL microcentrifuge tube.

7. Add 200 µL of Protein Precipitation Solution to the nuclear lysate. Draw the tube contents from the bottom of the tube, then expel the lysate rapidly down the side of the tube. Small protein clumps may be visible after mixing. Incubate on ice for 5 minutes.

8. Centrifuge at 13,000 × g for 10 minutes at room temperature. A greenish pellet should be visible. If any unpelleted debris is visible, repeat the centrifugation step.

9. Slowly transfer the supernatant to a clean 1.5 mL microcentrifuge tube by decanting the sample into a tube containing 600 µL of room-temperature isopropanol.
   Note: Some supernatant may remain in the original tube containing the protein pellet. Leave this residual liquid in the tube.

10. Gently mix the solution by gently inverting the tube eight times. Incubate for 1 minute at room temperature and repeat the inversion. White thread-like strands of DNA may form a visible mass.

11. Centrifuge at 16,000 × g for 2 minutes at room temperature. The DNA may be visible as a small white pellet.

12. Decant the supernatant and add 600 µL of room temperature 70% ethanol to the DNA. Gently invert the tube several times to wash the DNA pellet and the sides of the microcentrifuge tube. Centrifuge as instructed in Step 11.

13. Discard the supernatant then repeat Step 12.

14. Carefully aspirate the ethanol. The DNA pellet is very loose at this point so carefully avoid disturbing or aspirating the pellet into the pipette. Invert the tube on clean absorbent paper and air-dry the pellet for 10-15 minutes.

15. Add 100 µL of DNA Rehydration Solution to the tube. Do not vortex because this will cause mechanical shearing and decrease average fragment size. Rehydrate the DNA by incubating the solution overnight at room temperature.
