# Supplementary figures and images for: Combination of Sample Preservation Approaches and DNA Extraction Methods for Long‐Read Sequencing of Nudibranchs' Genomes
Source: Ecol Evol. 2025 Apr 15;15(4):e71262. doi: 10.1002/ece3.71262 (PMC11997370; doi:10.1002/ece3.71262)

## Nanodrop vs. Qubit concentration ratios

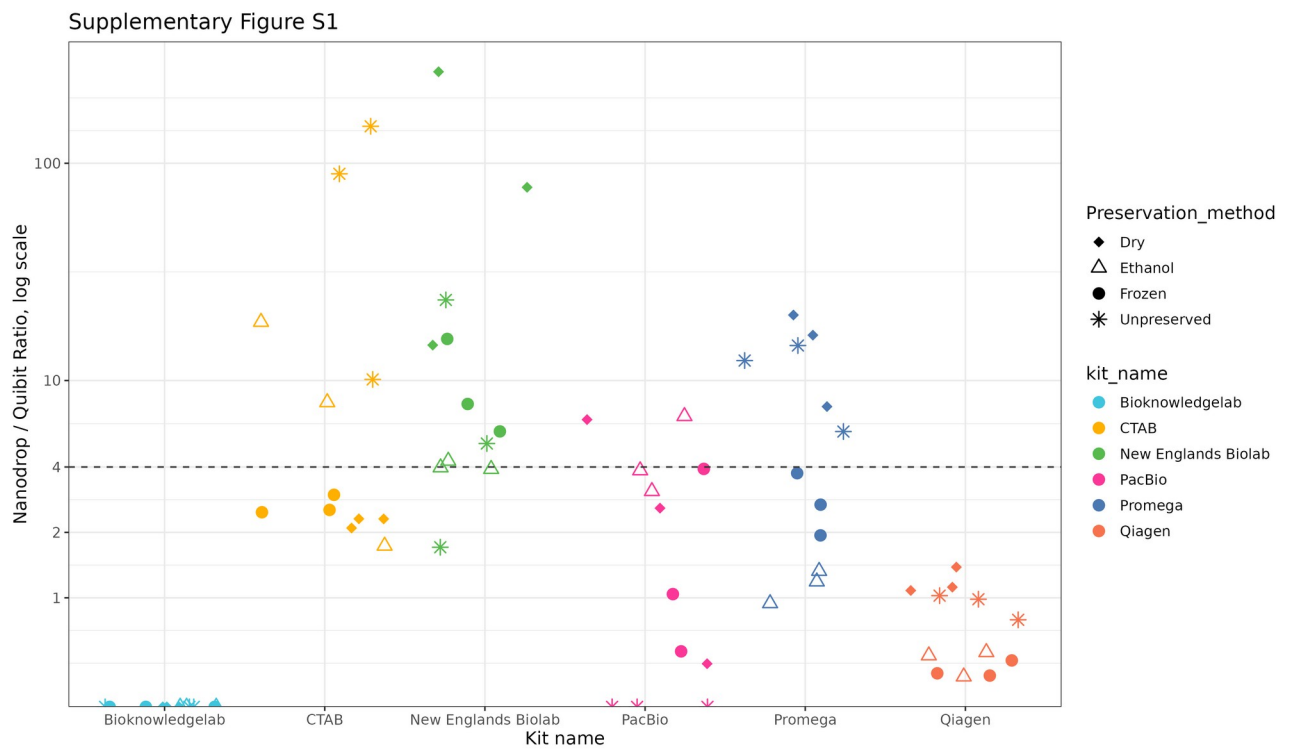

Supplement: Supplementary file 2 — Figure S1. [file ECE3-15-e71262-s001.pdf]
