# Supplementary material for: Combination of Sample Preservation Approaches and DNA Extraction Methods for Long‐Read Sequencing of Nudibranchs' Genomes
Source: Ecol Evol. 2025 Apr 15;15(4):e71262. doi: 10.1002/ece3.71262 (PMC11997370; doi:10.1002/ece3.71262)

Supporting Information

Figures S2

Fragment Analyzer results

Index

Initial DNA Extraction 2
Optimization of DNA extractions..4

Samples are named according to the kit identification letter: Bio Knowledge Lab (BKL), CTAB, New Englands Biolab (NEB), PacBio and Qiagen; followed by the preservation method: ethanol (E), Unpreserved (U), frozen (N) and fresh (F); and the replica, each numbered from 1 to 3.

Initial DNA Extraction

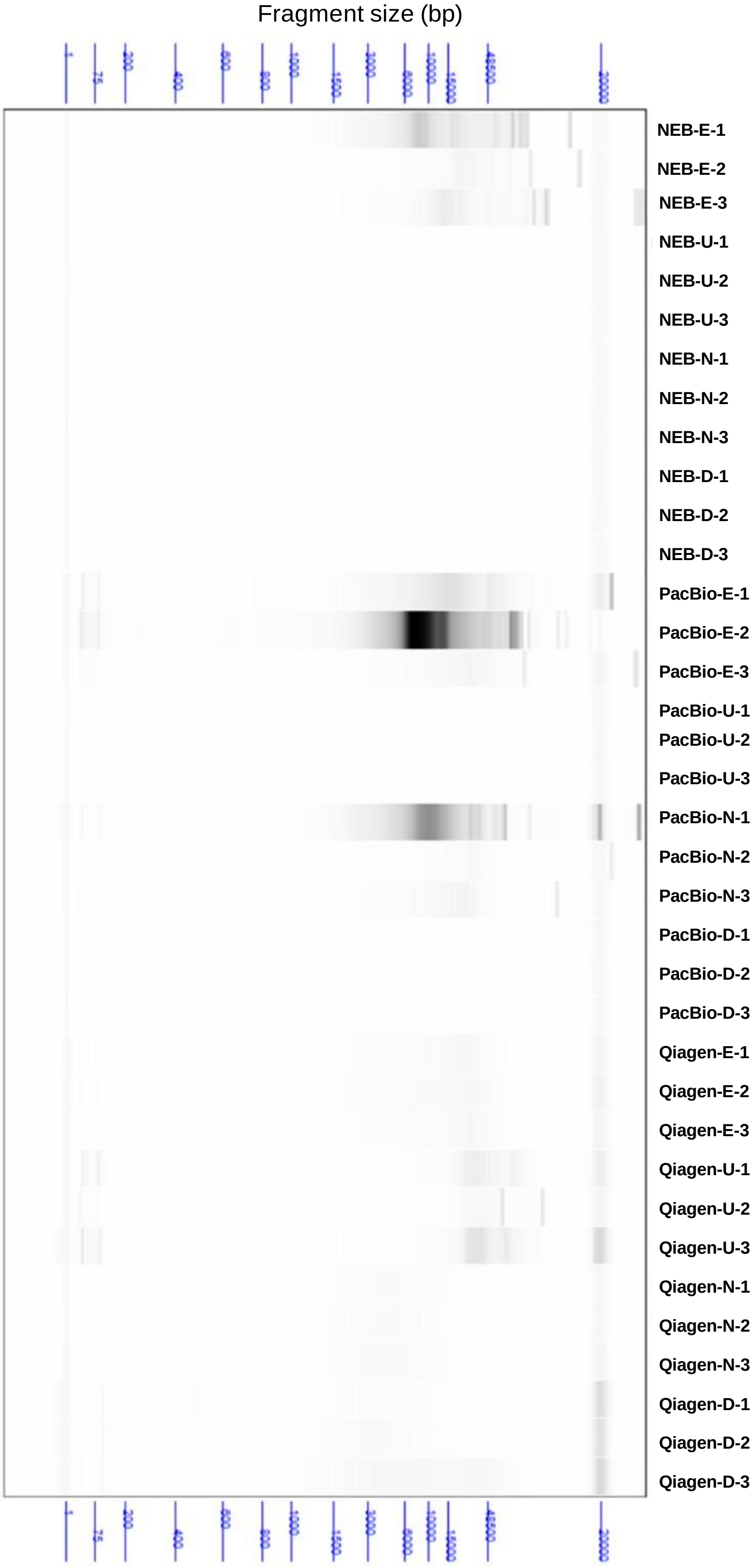

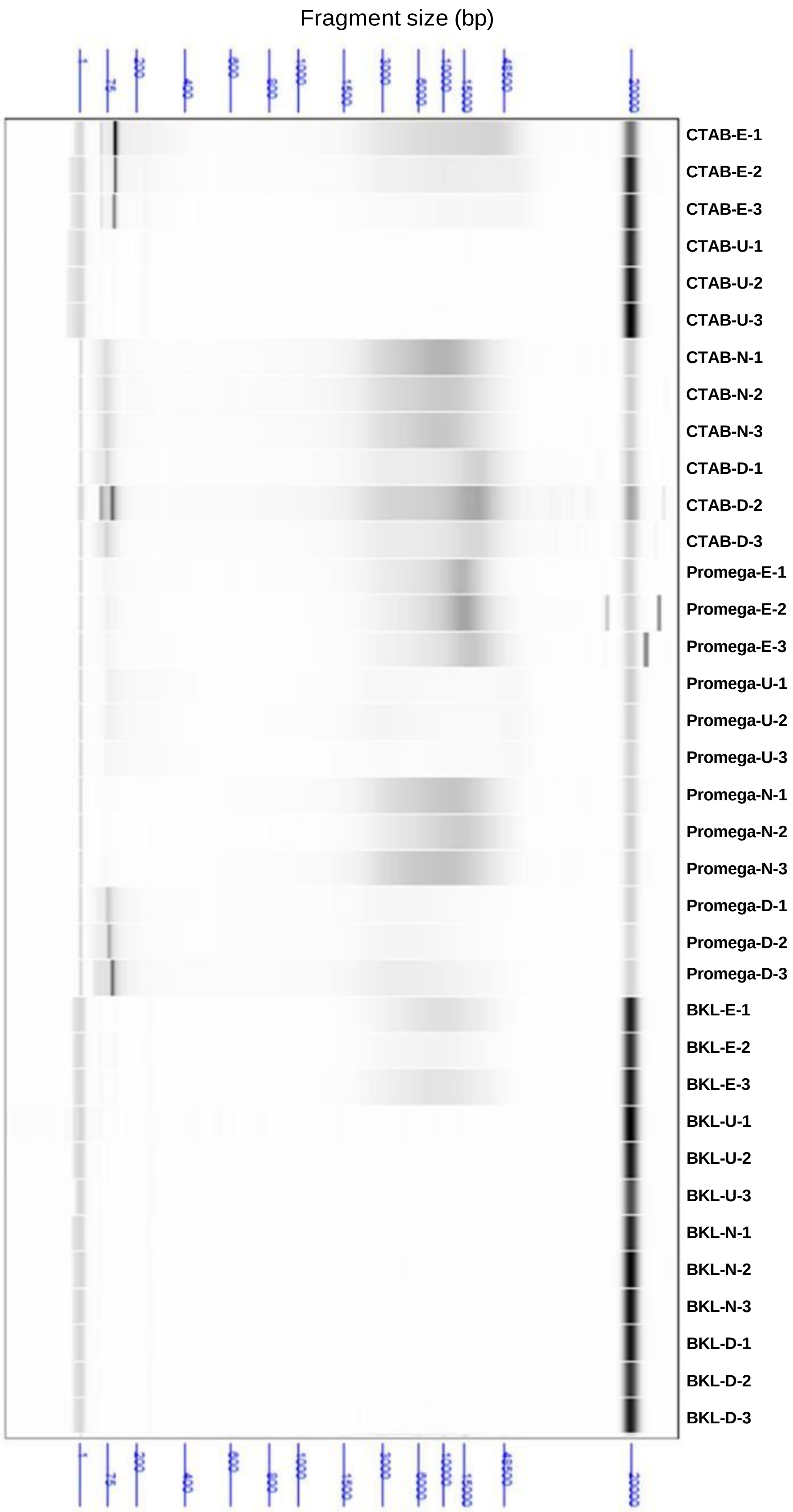
Fragment size (bp)
CTAB-E-1
CTAB-E-2
CTAB-E-3
CTAB-U-1
CTAB-U-2
CTAB-U-3
CTAB-N-1
CTAB-N-2
CTAB-N-3
CTAB-D-1
CTAB-D-2
CTAB-D-3
Promega-E-1
Promega-E-2
Promega-E-3
Promega-U-1
Promega-U-2
Promega-U-3
Promega-N-1
Promega-N-2
Promega-N-3
Promega-D-1
Promega-D-2
Promega-D-3
BKL-E-1
BKL-E-2
BKL-E-3
BKL-U-1
BKL-U-2
BKL-U-3
BKL-N-1
BKL-N-2
BKL-N-3
BKL-D-1
BKL-D-2
BKL-D-3

Optimization of DNA extractions

Fragment size (bp)

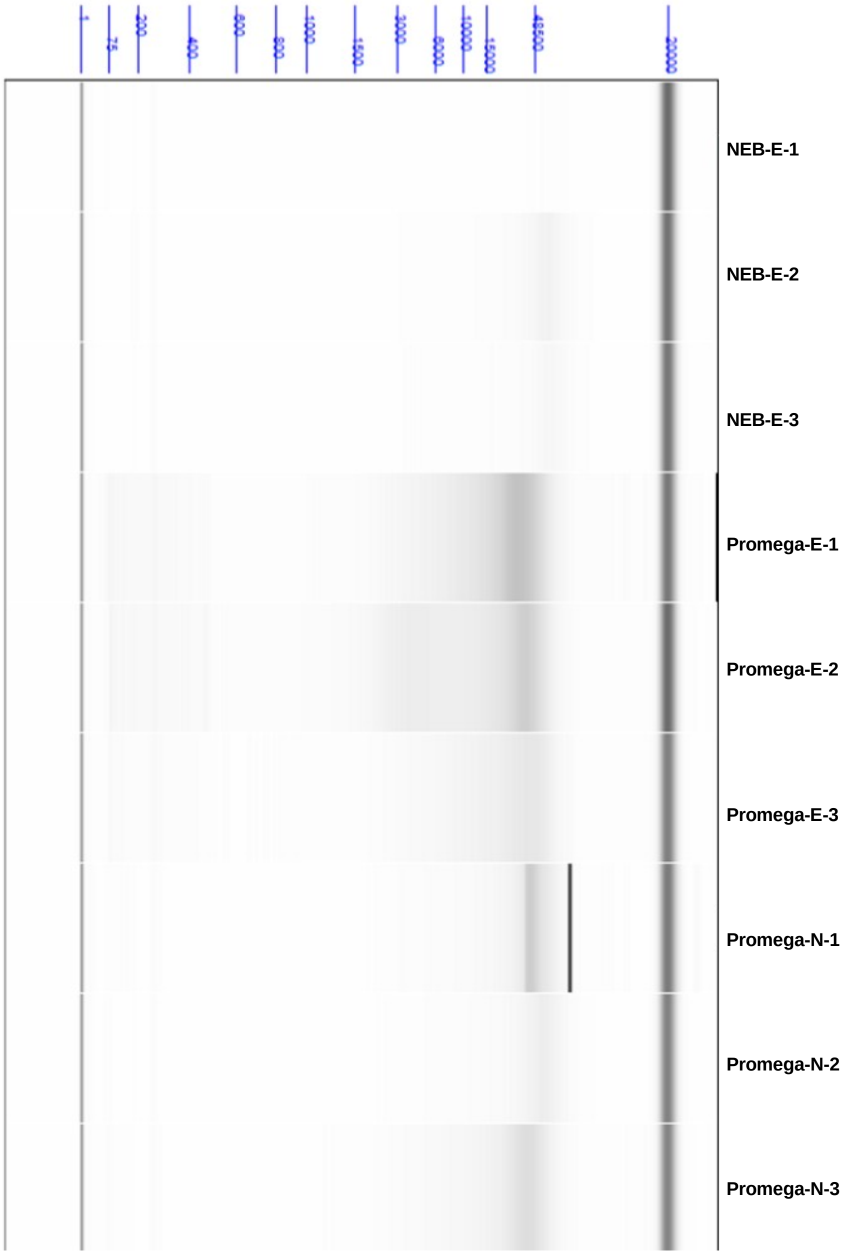

Supplement: Supplementary file 3 — Figure S2. [file ECE3-15-e71262-s002.pdf]
