# Supplementary material for: Combination of Sample Preservation Approaches and DNA Extraction Methods for Long‐Read Sequencing of Nudibranchs' Genomes
Source: Ecol Evol. 2025 Apr 15;15(4):e71262. doi: 10.1002/ece3.71262 (PMC11997370; doi:10.1002/ece3.71262)

Supporting Information
Figures S3

qPCR Melt curves

Index

BKL - Marine Animal Tissue Genomic DNA Extraction Kit (Bio Knowledge Lab, Ref: D2061) 2
CTAB - Cetyltrimethylammonium bromide (CTAB) protocol (Chakraborty et al., 2020) 2
NEB - MonarchⒹ HMW DNA Extraction Kit (New England Biolabs, Ref: T3060L) 3
PacBio - NanobindⒹ tissue kit RT (PacBio, Ref: 102-302-100) 3
Qiagen - Qiagen MagAttract HMW DNA Kit (REF: 67563) 4
Promega - Wizard HMW DNA Extraction Kit (REF: A2920) 5

BKL - Marine Animal Tissue Genomic DNA Extraction Kit (Bio Knowledge Lab, Ref: D2061)

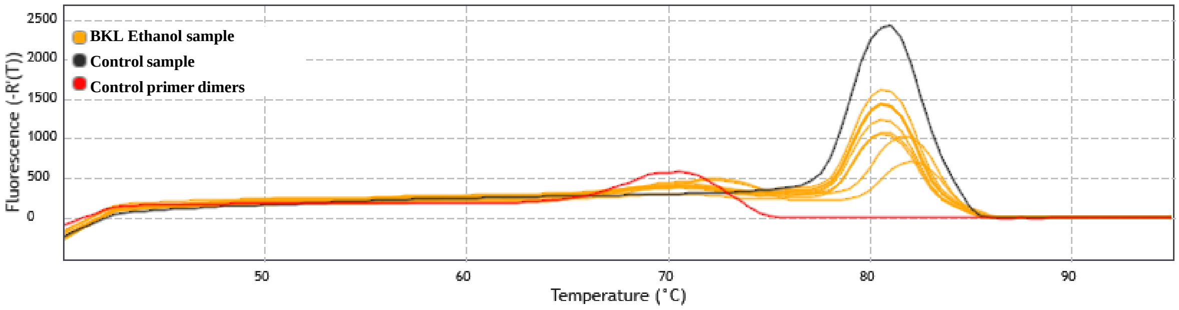

CTAB - Cetyltrimethylammonium bromide protocol (Chakraborty et al., 2020)

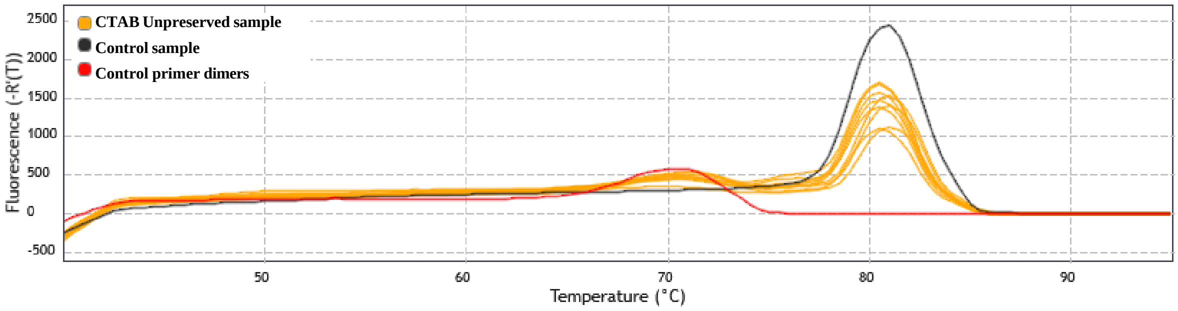

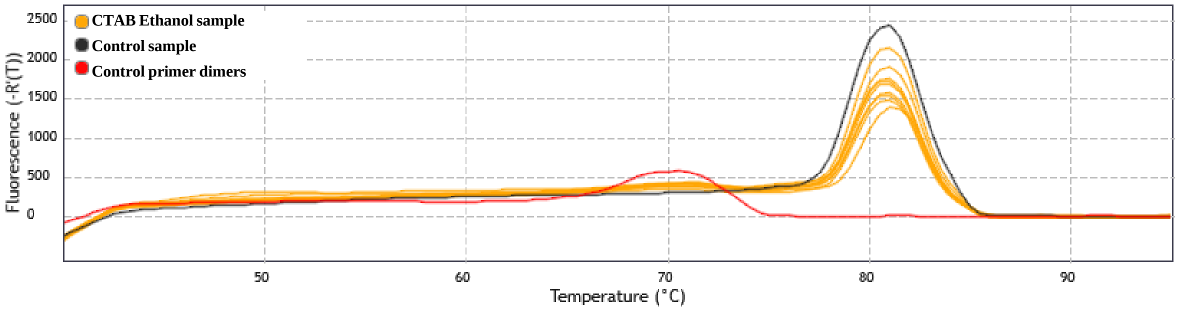

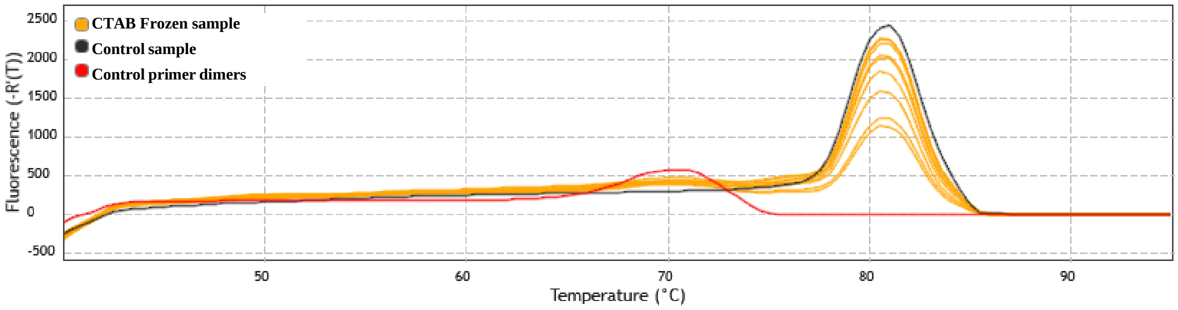

NEB – MonarchⒹ HMW DNA Extraction Kit (New England Biolabs, Ref: T3060L)

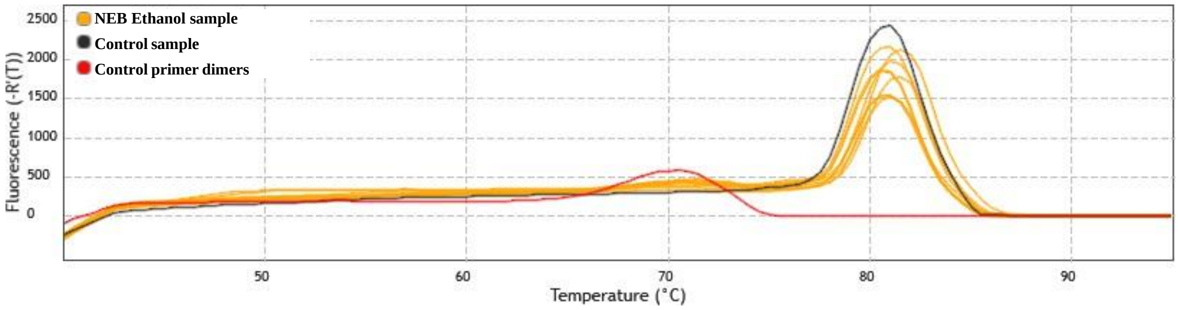

PacBio - NanobindⒹ tissue kit RT (PacBio, Ref: 102-302-100)

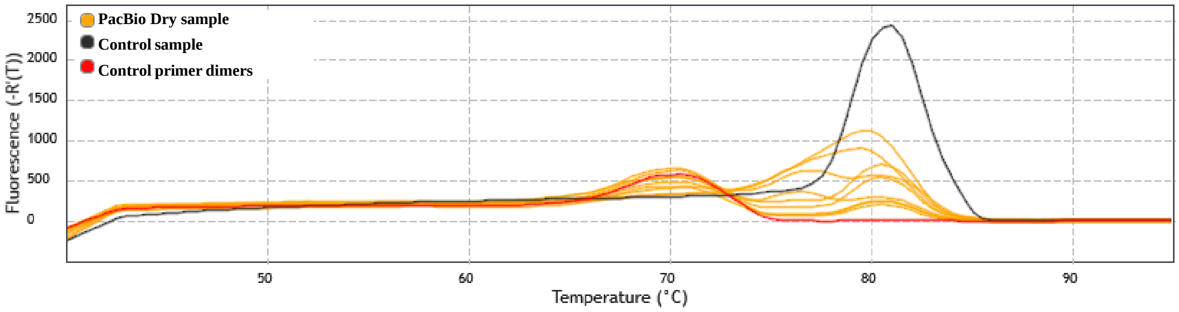

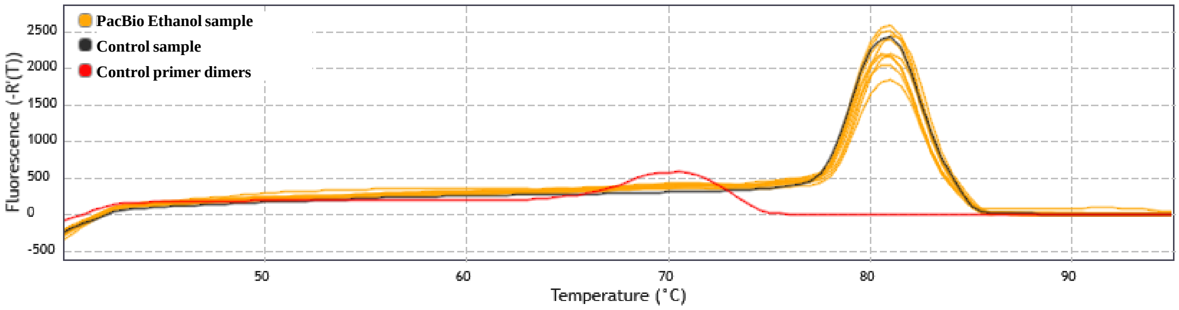

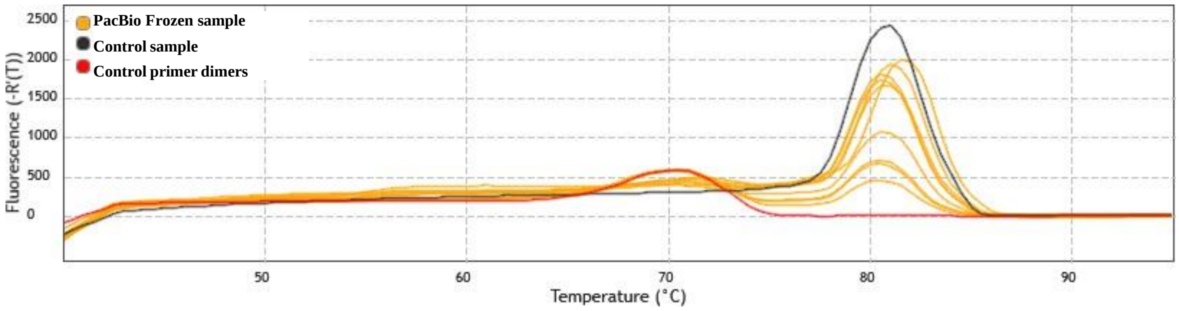

Qiagen - Qiagen MagAttract HMW DNA Kit (REF: 67563)

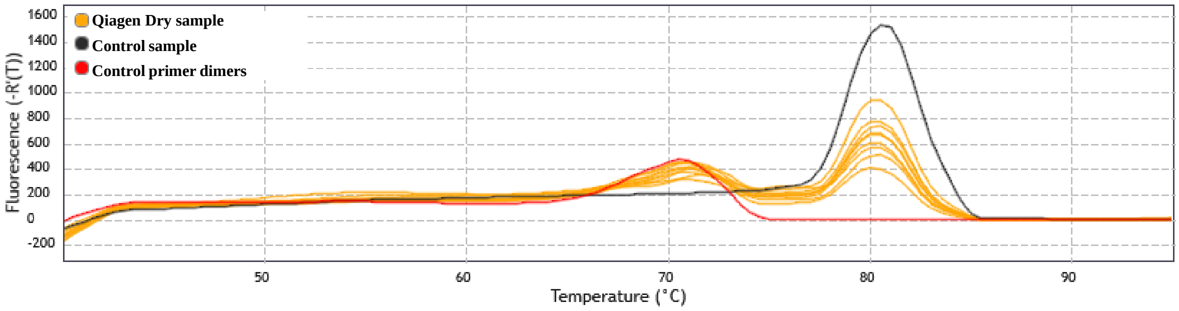

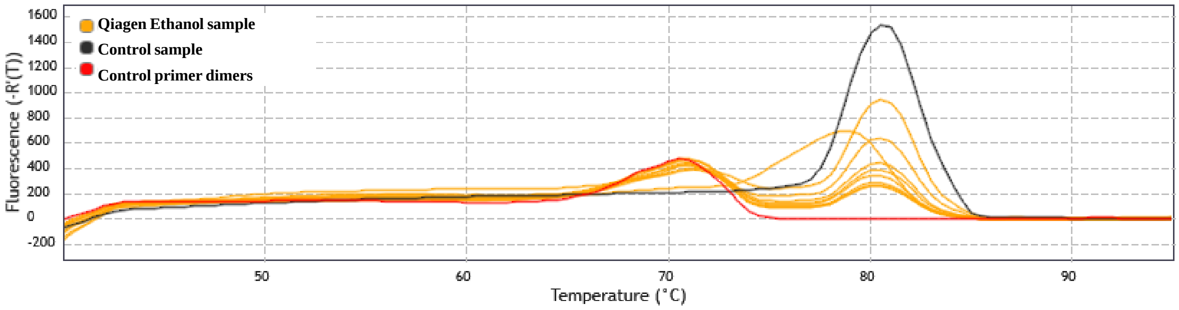

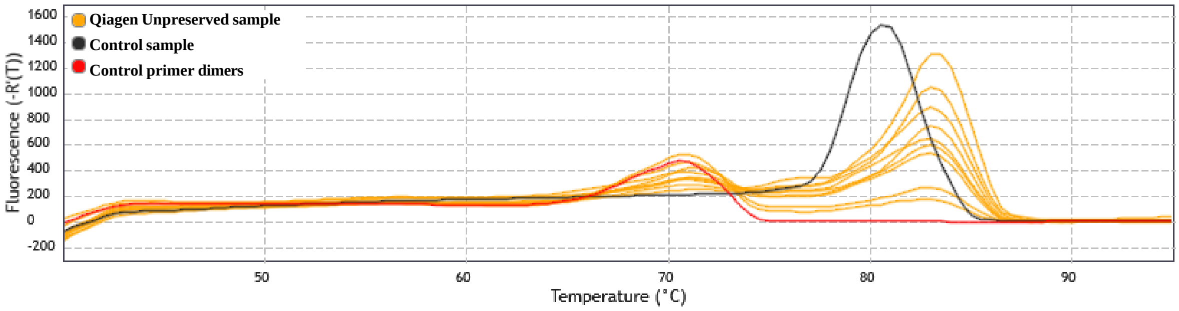

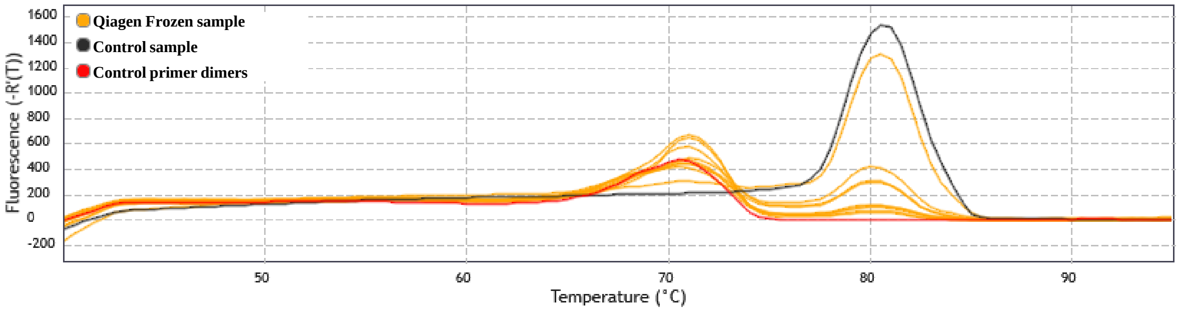

Promega - Wizard HMW DNA Extraction Kit (REF: A2920)

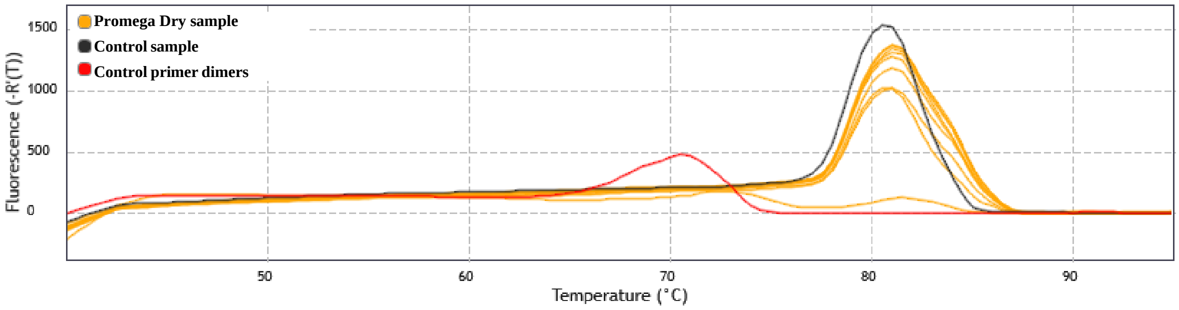

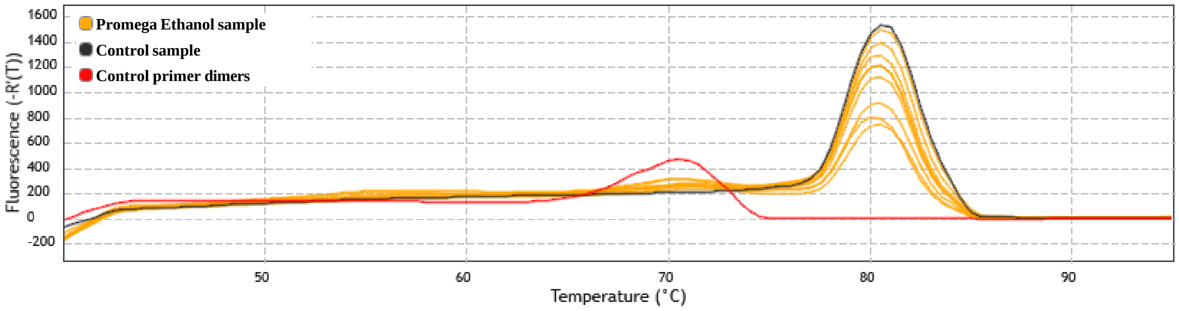

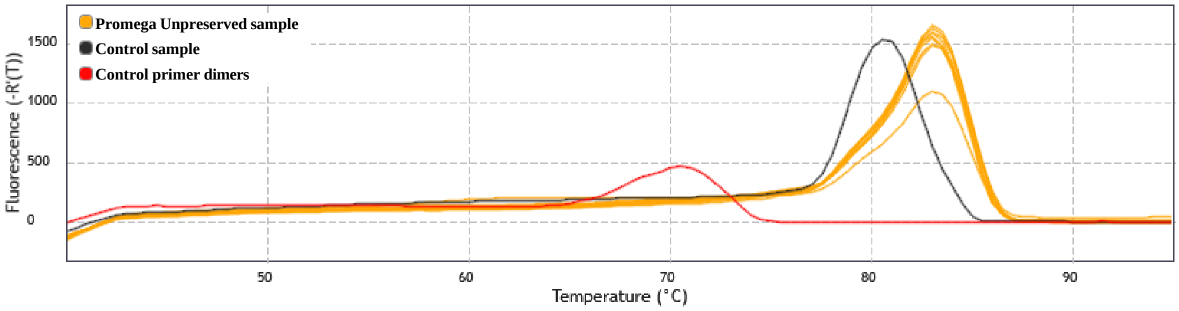

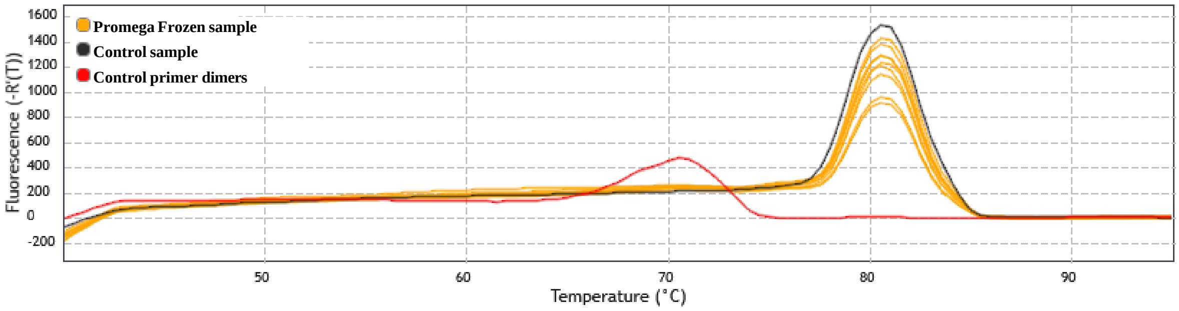

Supplement: Supplementary file 4 — Figure S3. [file ECE3-15-e71262-s005.pdf]
